# Second-line treatments for Advanced Hepatocellular Carcinoma: A Systematic Review and Bayesian Network Meta-analysis

**DOI:** 10.1007/s10238-021-00727-7

**Published:** 2021-06-19

**Authors:** Antonio Giovanni Solimando, Nicola Susca, Antonella Argentiero, Oronzo Brunetti, Patrizia Leone, Valli De Re, Rossella Fasano, Markus Krebs, Elisabetta Petracci, Irene Azzali, Oriana Nanni, Nicola Silvestris, Angelo Vacca, Vito Racanelli

**Affiliations:** 1 Guido Baccelli Unit of Internal Medicine, Department of Biomedical Sciences and Human Oncology, School of Medicine, Aldo Moro University of Bari, Bari, Italy; 2 IRCCS Istituto Tumori Giovanni Paolo II of Bari, Bari, Italy; 3 Bio-Proteomics Facility, Department of Translational Research, Centro Di Riferimento Oncologico Di Aviano (CRO) IRCCS, Aviano, Italy; 4 Department of Biomedical Sciences and Human Oncology, School of Medicine, Aldo Moro University of Bari, Bari, Italy; 5 Comprehensive Cancer Center Mainfranken, University Hospital Würzburg, Würzburg, Germany; 6 Unit of Biostatistics and Clinical Trials, Istituto Scientifico Romagnolo Per Lo Studio E La Cura Dei Tumori (IRST) IRCCS, Meldola, Italy

**Keywords:** Hepatocellular carcinoma, Second-line treatment, Network meta-analysis

## Abstract

**Background & Aims:**

A plethora of second-line therapies have been recently introduced for hepatocellular carcinoma (HCC) treatment with promising results. A meta-analysis of second-line treatments for HCC has been performed to better tailor their use based on improved patient stratification and to identify the best available option.

**Methods:**

Pubmed, Scopus, Web of Science, and ClinicalTrials.gov were searched for randomized controlled trials evaluating second-line treatment for advanced HCC in patients already treated with sorafenib. The primary outcome was overall survival (OS). Secondary outcomes were progression-free survival (PFS) and drug withdrawal due to adverse events. Network meta-analyses were performed considering placebo as the basis for comparison in efficacy and safety analyses. Subgroup stratification considered gender, age, sorafenib-responsiveness and drug tolerability, viral infection, macrovascular invasion, HCC extrahepatic spread, performance status, and alpha-fetoprotein levels.

**Results:**

Fourteen phase II or III randomized controlled trials, involving 5,488 patients and 12 regimens, were included in the analysis. Regorafenib (hazard ratio (HR) = 0.63, 95% confidence interval (CI) = 0.50–0.79), cabozantinib (HR = 0.76, 95% CI = 0.63–0.92), and ramucirumab (HR = 0.82, 95% CI = 0.70–0.76) significantly prolonged OS compared with placebo. Cabozantinib (HR = 0.44, 95% CI = 0.36–0.52), regorafenib (HR = 0.46, 95% CI = 0.37–0.56), ramucirumab (HR = 0.54, 95% CI = 0.43–0.68), brivanib (HR = 0.56, 95% CI = 0.42–0.76), S-1 (HR = 0.60, 95% CI = 0.46–0.77), axitinib (HR = 0.62, 95% CI = 0.44–0.87), and pembrolizumab (HR = 0.72, 95% CI = 0.57–0.90) significantly improved PFS compared with placebo. None of the compared drugs deemed undoubtedly superior after having performed a patients’ stratification.

**Conclusions:**

The results of this network meta-analysis suggest the use of regorafenib and cabozantinib as second-line treatments in HCC.

**Supplementary Information:**

The online version contains supplementary material available at 10.1007/s10238-021-00727-7.

## Introduction

Hepatocellular carcinoma (HCC) is one of the most common cancers and the fourth leading cause of cancer-related death worldwide [1][2]*.* As a global disease, HCC differs regionally in its etiology, ethnic distribution, and treatment [3][4].

Most patients (70–80%) are diagnosed with advanced disease unsuitable for locoregional treatment and thus mostly receive palliative care [5][6]. According to the Barcelona Clinic Liver Cancer (BCLC) staging, advanced HCC is classified as C-HCC and includes vascular involvement/extrahepatic spread, physical impairment, and Eastern Cooperative Oncology Group Performance Status (ECOG PS) 1–2 [7].

Because HCC is highly chemorefractory, systemic therapeutic strategies often fail. Sorafenib, a small multikinase inhibitor, was one of the first drugs to improve disease outcome in patients with advanced HCC and was approved by the FDA (Food and Drug Administration) in 2007. However, overall survival (OS) in treated patients is less than one year [8]. After more than 10 years, the REFLECT trial showed the non-inferiority of lenvatinib over sorafenib in a head-to-head study of selected patients with treatment-naive advanced HCC, ECOG PS 0–1, ≤ 50% liver involvement, and without invasion of the bile duct or a main branch of the portal vein [9]. More recently, encouraging antitumor activity was obtained using a combination of anti-antiangiogenic agents with immunotherapy. This has led to the approved use of atezolizumab plus bevacizumab as a first-line treatment for patients with unresectable HCC. The IMbrave 150 study showed better OS and progression-free survival (PFS) outcomes of this new combination therapy than achieved with sorafenib [10]. The OS at 12 months was 67.2% (95% confidence interval [CI], 61.3–73.1) with atezolizumab–bevacizumab and 54.6% (95% CI, 45.2–64.0) with sorafenib.

Likewise, second-line treatment has also evolved in an attempt to address an important unmet clinical need for drugs that are more effective than the best supportive care (BSC). The phase III studies RESORCE, CELESTIAL, and REACH reported clinical benefits of regorafenib, cabozantinib, and ramucirumab, respectively, over placebo in patients pretreated with sorafenib. Immune checkpoint inhibitors have also been examined as novel second-line agents in the treatment of HCC [11][12]. They include nivolumab (alone or in combination with ipilimumab, an anti-CTLA-4 monoclonal antibody) and pembrolizumab, two monoclonal antibodies that block the programmed death-1 (PD-1) pathway and have been approved by the FDA for already treated HCC, following promising results in the CheckMate 040 and KEYNOTE-224 clinical trials [13][14][15].

Despite substantial improvements in the OS of patients with advanced HCC due to the availability of more effective treatments, only 40–50% of patients undergo second-line treatment [9]. While two studies support the use of regorafenib in patients with advanced or intermediate HCC ineligible for locoregional treatment, Child–Pugh class A disease, and disease progression after first-line sorafenib therapy [16][17], the quality of the evidence is constrained by the following caveats. First, 40% of the patients enrolled in the trial were Asian; whether the results can be generalized to the Caucasian population is unclear. Second, a small number of adverse events were reported, which affected the accuracy of the results. Third, whether regorafenib is safe and effective in sorafenib-intolerant subjects is unknown, as these patients were not included in the phase III study [16][17].

Therefore, given the considerable complexity of the therapeutic landscape [2], the aim of this meta-analysis is to compare the efficacy and safety of second‐line agents and to highlight the strengths and weaknesses of the available clinical data. Moreover, this study points toward a personalized approach based on novel criteria for the second-line treatment of HCC.

## Methods

This systematic review and network meta-analysis were conducted according to the guidelines of the PRISMA extension statement for the reporting of systematic reviews incorporating network meta-analyses of health care interventions. The protocol of this study was not registered.

## Search strategy and selection criteria

A literature search in Pubmed, Scopus, Web of Science, and ClinicalTrials.gov databases was undertaken from the inception of each database to December 31, 2020, using the following search string: “(hepatocarcinoma OR hepatocellular carcinoma) AND (second-line OR refractory) AND (trial).” Abstracts and presentations from all major conference proceedings, including the American Society of Clinical Oncology and the European Society for Medical Oncology, until December 31, 2020, were also reviewed.

The identified reports were independently screened by two investigators (N.Su. and A.G.S) by title and Abstract to confirm that they adhered to the eligibility criteria listed below. In case of disagreement, a third investigator (A.A.) was involved. Potentially relevant reports were subjected to a full-text assessment to determine their compliance with the criteria for inclusion in the systematic review and meta-analysis.

The inclusion criteria for studies included in the review and meta-analysis followed the PICO system: 1) patients 18 years of age or older with advanced HCC already treated with sorafenib; 2) patients who received a second-line systemic treatment in a phase 2 or phase 3 controlled clinical trial; 3) OS, PFS, and drug withdrawal due to adverse events. To avoid excessive heterogeneity, single-arm phase 1 and 2 trials were excluded as were studies without a placebo-controlled arm. To improve the methodological framework of this study, only peer-reviewed reports were included. In addition, eligibility was limited to English-language publications.

## Data extraction and outcome measures

The data were independently extracted by N.Su. and A.G.S. to improve the subgroup analysis. Secondary sources were screened to obtain additional information regarding subgroups (supplementary material in published articles and the ClinicalTrials.gov study page). The following data were extracted for each study: name of trial, trial registration number, year of publication, randomization, blinding, and number of patients. Data expressed as the hazard ratio (HR) and 95% confidence interval (CI) were extracted to assess OS (main outcome) and PFS. The data frequency was first determined and the odds ratio (ORs) and 95% CI then calculated to assess drug withdrawal due to adverse events (as a proxy for toxicity). Time to progression was used as a proxy for PFS in two studies, because the latter was not provided. The studies were analyzed according to intention-to-treat, and the assessment of tumor progression was based upon RECIST (v 1.1) for all trials, excepted that published by Llovet et al. [18], in which mRECIST criteria were considered. Data for adverse events were not extracted because of inter-study inconsistencies in their reporting. Treatment rankings were obtained as described below. Placebo was chosen as the common basis of comparison in the primary analyses. In the analysis of the CELESTIAL trial [19], the subgroup of patients exposed to a single first-line treatment was also considered, to avoid biases related to a more advanced HCC stage.

## Additional analyses

Subgroup analysis for OS was performed for: male/female patients, young/old patients, sorafenib refractory/intolerant patients, HBV/HCV/non-infected patients, patients with/without macrovascular invasion, patients with/without extrahepatic spread, ECOG PS 0/ECOG PS > 0, and high/low alpha-fetoprotein (AFP) levels. The cut-off for the two age subgroups was 65 years, except in one study [20], in which it was 60 years. The cut-off for the AFP level was 200 ng/mL in four studies [18][21][22][23], 200 IU/mL in one study [24], and 400 ng/mL in five studies [16][19][25][26][27]. One study of tivantinib reported AFP-stratified HRs with a cut-off of 144 ng/mL [28]. For two studies [29][30], a subgroup analysis could not be performed.

Regorafenib was chosen as the basis for comparison in the additional analyses of survival because it was determined to be the best treatment in the overall population included in this network meta-analysis and had an impact on OS across all patient subgroups. The stratified analysis was applied to the other treatments according to the subgroups reported in the RESOURCE trial.

## Bias assessment

The risk of bias within studies was assessed using the Cochrane Risk of Bias Tool.

## Statistical analysis

The network meta-analysis was performed within a frequentist framework using the graph theoretical method. Network connectivity was visually assessed in a network plot. As none of the studies included direct comparisons between treatments, an inconsistency analysis could not be performed. Instead, the total heterogeneity of the network was attributable only to within-design variation. Since the Q-statistic corresponding to within-design heterogeneity was not significant (p = 0.589), the results using a fixed-effects or random-effects model were identical. Therefore, in this analysis a fixed-effect model was used.

In the overall sample and for each of the subgroups analyses, treatments were ranked employing the P-score, a frequentist analog of SUCRA, as described by Rücker & Schwarzer [31].

All statistical analyses were performed using the R statistical language (R version 3.6.2, release date: 2019–12-12; R Foundation for Statistical Computing, Vienna, Austria).

## Results

### Systematic review and study characteristics

**Fig. 1:**
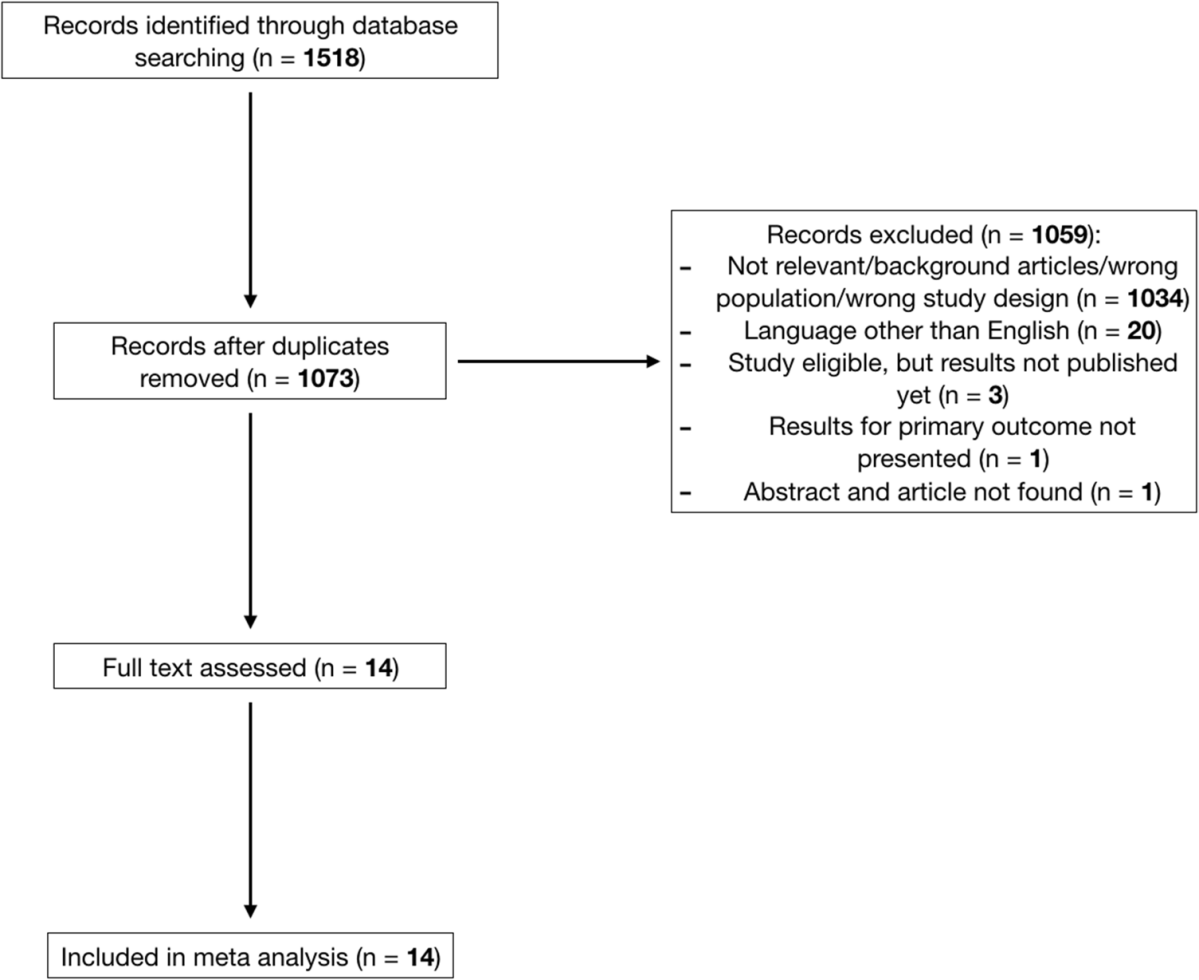
PRISMA flow diagram

The database search yielded 1518 results. After duplicates removal, 1073 records were screened through titles and Abstracts. Fourteen records were selected for full-text assessment and all were included in the quantitative analysis [16][18][19][20][28][24][22] [29][30][21][26][27][23][25] (Fig. 1). The 14 studies were randomized controlled trials (RCTs), with a total population of 5488 patients (3568 in the active treatment groups and 1920 in the placebo group). The network comprised 12 arms (11 active treatments and placebo) (Supplementary Fig. 1). The drugs tested in the active treatments were ADIPEG20, axitinib, brivanib, cabozantinib, codrituzimab, everolimus, pembrolizumab, ramucirumab, regorafenib, S-1, and tivantinib. No trials directly compared different active treatments. The characteristics of the studies and their populations are summarized in Table 1 and Supplementary Table 1, respectively.

**Table 1:** Summary of the included studies

Study name	Year	Trial registration number	Blinding	Randomization (ratio)	Phase	Participants	Active arm (n)	Basis of comparison (number of studies)
BRISK-PS [18]	2013	NCT00825955	Double	Yes (2:1)	3	395	Brivanib (263)	Placebo (132)
Santoro A. et al. [24]	2013	NCT00988741	Double	Yes (2:1)	2	107	Tivantinib (71)	Placebo (36)
EVOLVE-1 [21]	2014	NCT01035229	Double	Yes (2:1)	3	546	Everolimus (362)	Placebo (184)
Kang Y.-K. et al. [25]	2015	NCT01210495	Double	Yes (2:1)	2	202	Axitinib (134)	Placebo (68)
REACH [26]	2015	NCT01140347	Double	Yes (1:1)	3	565	Ramucirumab (283)	Placebo (282)
Abou-Aifa G.K. et al. [29]	2016	NCT01507168	Double	Yes (2:1)	2	185	Codrituzumab (125)	Placebo (60)
RESORCE [16]	2017	NCT01774344	Double	Yes (2:1)	3	573	Regorafenib (379)	Placebo (194)
S-CUBE [30]	2017	JapicCTI-090920	Double	Yes (2:1)	3	333	S-1 (222)	Placebo (111)
Abou-Aifa G. et al. [20]	2018	NCT01287585	Double	Yes (2:1)	3	635	ADIPEG20 (424)	Placebo (211)
CELESTIAL [19]	2018	NCT01908426	Double	Yes (2:1)	3	707	Cabozantinib (470)	Placebo (237)
Metiv-HCC [22]	2018	NCT01755767	Double	Yes (2:1)	3	340	Tivantinib (226)	Placebo (114)
REACH 2 [27]	2019	NCT02435433	Double	Yes (2:1)	3	292	Ramucirumab (197)	Placebo (95)
JET-HCC [28]	2020	NCT02029157	Double	Yes (2:1)	3	195	Tivantinib (134)	Placebo (61)
KEYNOTE-240 [23]	2020	NCT02702401	Double	Yes (2:1)	3	413	Pembrolizumab (278)	Placebo (135)

## Risk of bias analysis

The results of the risk of bias analysis within studies are reported in Supplementary Table 2. All 14 RCTs compared active treatment with placebo. The overall quality of the studies was high, although only two provided sufficient detail on allocation concealment. Moreover, some studies were at risk of performance or detection bias due to a lack of blinding of the care-provider or outcomes assessor. Six studies were at high risk of reporting bias as they did not provide information on OS stratified for the subgroups that were of interest for the purpose of this network meta-analysis.

## Overall survival

**Fig. 2:**
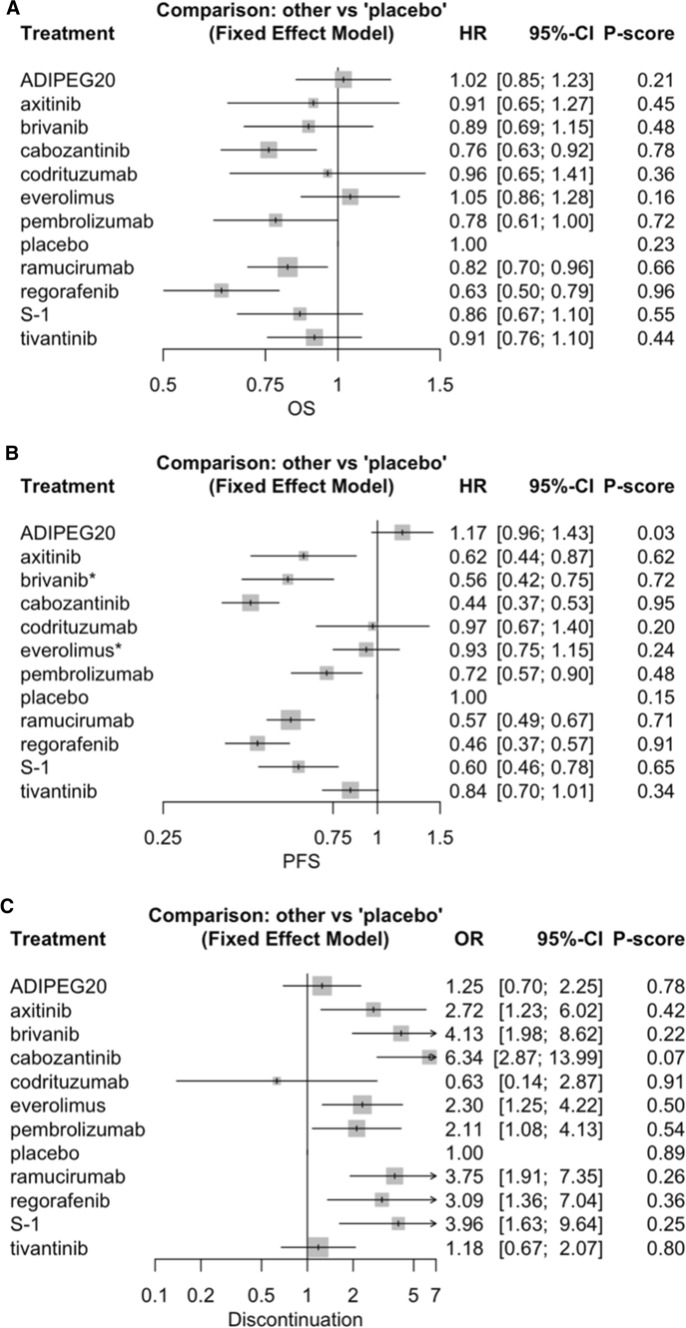
A: Forest plot of overall survival. The comparisons were made against the placebo; B: Forest plot of progression-free survival. The comparisons were made against the placebo; C: Forest plot of the discontinuation of active treatment. The comparisons were made against regorafenib

The pooled results across all groups suggested a greater OS benefit from regorafenib (HR 0.63, 95% CI 0.50–0.79), cabozantinib (HR 0.76, 95% CI 0.63–0.92), and ramucirumab (HR 0.82, 95% CI 0.70–0.76) vs. placebo, with the results for pembrolizumab nearly reaching statistical significance (HR 0.78, 95% CI 0.61–1.00). There was no substantial difference in the results after the exclusion of patients receiving cabozantinib as third-line treatment (HR 0.74, 95% CI 0.59–0.92) (Table 2; Fig. 2A). There were also no statistically significant differences between treatments compared to regorafenib, excluding ADIPEG20 (HR 1.62, 95% CI 1.21–2.18), everolimus (HR 1.67, 95% CI 1.23–2.25), placebo (HR 1.59, 95% CI 1.26–2.00), and tivantinib (HR 1.45, 95% CI 1.08–1.94), all of which nearly reached statistical significance for inferiority (Supplementary Fig. 2).

**Table 2:** Main outcomes of the studies included in the meta-analysis

Study name	Study name	OS	PFS	Treatment discontinuation
BRISK-PS [18]	BRISK-PS	0.89 (0.69–1.15)	0.56 (0.42–0.76)*	4.13 (1.98–8.62)
Santoro A. et al. [24]	Tivantinib	0.90 (0.57–1.40)	0.67 (0.44–1.04)	0.78 (0.29–2.11)
EVOLVE-1 [21]	EVOLVE-1	1.05 (0.86–1.27)	0.93 (0.75–1.15)*	2.30 (1.26–4.24)
Kang Y.-K. et al. [25]	Axitinib	0.91 (0.65–1.27)	0.62 (0.44–0.87)	2.72 (1.23–6.02)
REACH [26]	REACH	0.87 (0.72–1.05)	0.63 (0.52–0.75)	3.77 (1.69–8.42)
Abou-Aifa G.K. et al. [29]	Codrituzumab	0.96 (0.65–1.41)	0.97 (0.67–1.39)	0.63 (0.14–2.90)
RESORCE [16]	RESORCE	0.63 (0.50–0.79)	0.46 (0.37–0.56)	3.09 (1.36–7.05)
S-CUBE [30]	S-CUBE	0.86 (0.67–1.10)	0.60 (0.46–0.77)	3.96 (1.63–9.65)
Abou-Aifa G. et al. [20]	ADIPEG20	1.02 (0.85–1.23)	1.18 (0.96–1.43)	1.25 (0.70–2.26)
CELESTIAL [19]	CELESTIAL	0.76 (0.63–0.92)	0.44 (0.36–0.52)	6.34 (2.87–13.98)
Metiv-HCC [22]	METIV-HCC	0.97 (0.75–1.25)	0.96 (0.75–1.22)	1.33 (0.64–2.68)
REACH 2 [27]	REACH 2	0.71 (0.53–0.95)	0.45 (0.34–0.60)	3.70 (1.08–12.74)
JET-HCC [28]	JET-HCC	0.82 (0.58–1.15)	0.74 (0.52–1.04)	2.83 (0.33–24.07)
KEYNOTE-240 [23]	KEYNOTE-240	0.78 (0.61–1.00)	0.72 (0.57–0.90)	2.11 (1.08–4.13)

Trials outcomes of active treatment vs placebo are expressed as the hazard ratio and 95% confidence interval for overall survival (OS) and progression-free survival (PFS) and as the odds ratio and 95% confidence interval for treatment discontinuation

^*^Time to progression used instead of PFS

## Progression-free survival

A significantly prolonged PFS compared to placebo was determined for cabozantinib (HR 0.44, 95% CI 0.36–0.52), regorafenib (HR 0.46, 95% CI 0.37–0.56), ramucirumab (HR 0.54, 95% CI 0.43–0.68), brivanib (HR 0.56, 95% CI 0.42–0.76), S-1 (HR 0.60, 95% CI 0.46–0.77), axitinib (HR 0.62, 95% CI 0.44–0.87), and pembrolizumab (HR 0.72, 95% CI 0.57–0.90) (Table 2; Fig. 2B). However, none of these drugs had statistically significant superiority when compared to regorafenib (Supplementary Fig. 3).

## Overall survival subgroup analyses

Table 3 shows the first, second, and last ranked treatments for each subgroup. Regorafenib ranked first for patients with the following characteristics: low-age, male, ECOG PS 0, presence of extrahepatic spread, HBV, HCV, low AFP, high AFP, and progression while on sorafenib. Cabozantinib ranked first for older patients and without HBV/HCV infection individuals. Tivantinib was as good as regorafenib in females and in HCV-infected patients. Axitinib ranked first in ECOG PS > 0 patients, and pembrolizumab in patients with macrovascular invasion. Ramucirumab was equivalent to regorafenib in patients with high AFP levels. Brivanib ranked first in sorafenib-intolerant patients but these results were not statistically significant. Overall, no compound exhibited clinical efficacy in the sorafenib-intolerant subgroup of patients (Supplementary Fig. 4a). Of note, in the regorafenib trial [16] this subgroup was not included, whereas in the cabozantinib trial [19] the subgroup analysis was not available.

**Table 3:** Treatment ranking by group (P-score)

	Treatment		
Group	Best	Alternative	Worst
Overall (OS)	Regorafenib (0.96)	Cabozantinib (0.78)	Everolimus (0.16)
Overall (PFS)	Cabozantinib (0.90)	Regorafenib (0.87)	ADIPEG20 (0.07)
Overall (discontinuation)	Codrituzumab (0.91)	Placebo (0.89)	Cabozantinib (0.07)
Age			
Low	Regorafenib (0.88)	Axitinib (0.73)	ADIPEG20 (0.07)
High	Cabozantinib (0.81)	Regorafenib (0.80)	Everolimus (0.20)
Sex			
Female	Tivantinib (0.75)	Cabozantinib (0.68)	Placebo (0.30)
Male	Regorafenib (0.91)	Pembrolizumab (0.71)	Placebo (0.20)
ECOG			
0	Regorafenib (0.92)	Cabozantinib (0.80)	Axitinib (0.20)
> 0	Axitinib (0.88)	Regorafenib (0.65)	Placebo (0.25)
Extrahepatic spread	Regorafenib (0.97)	Cabozantinib (0.80)	ADIPEG20 (0.19)
Macrovascular invasion	Pembrolizumab (0.85)	Regorafenib (0.79)	ADIPEG20 (0.17)
HBV infection	Regorafenib (0.82)	Pembrolizumab (0.81)	Placebo (0.13)
HCV infection	Regorafenib/Tivantinib (0.71)	Brivanib (0.69)	ADIPEG20 (0.14)
No HBV/HCV infection	Cabozantinib (0.83)	Ramucirumab (0.68)	Everolimus (0.06)
AFP			
Low	Regorafenib (0.85)	Pembrolizumab (0.83)	Axitinib (0.12)
High	Ramucirumab/Regorafenib (0.78)	Axitinib (0.76)	Everolimus (0.05)
Sorafenib intolerance	Brivanib (0.62)	Ramucirumab (0.60)	Everolimus (0.32)
Sorafenib progression	Regorafenib (0.96)	Pembrolizumab (0.79)	ADIPEG20 (0.11)

The pooled results across the subgroup with disease progression despite sorafenib treatment suggested that OS was better in patients treated with regorafenib (HR 0.63, 95% CI 0.50–0.79), pembrolizumab (HR 0.73, 95% CI 0.56–0.95), and ramucirumab (HR 0.81, 95% CI 0.69–0.97) (Supplementary Fig. 4b). The lack of data for cabozantinib-treated patients prevented a similar analysis.

Detailed forest plots for the OS subgroups analysis are provided in Appendix 1. Supplementary Table 3 shows the HRs for OS stratified by subgroup as reported in the trials considered in this analysis.

## Treatment discontinuation

All therapies except ADIPEG20, codrituzumab, and tivantinib were associated with a higher risk of discontinuation due to treatment-related adverse events compared to placebo. The treatment ranking in this analysis was, from worst to best: cabozantinib, brivanib, S-1, ramucirumab, regorafenib, axitinib, everolimus, pembrolizumab, ADIPEG20, tivantinib, placebo, and codrituzumab. Only cabozantinib had a statistically significant higher OR of treatment discontinuation compared to placebo (OR 6.34, 95% CI 2.87–13.99) (Table 2, Fig. 2c).

## Discussion

Despite a better understanding of the pathogenesis of HCC, for patients with advanced disease there are few therapeutic options, and the prognosis remains poor. Sorafenib still represents a mainstay of treatment and is often the first-line therapy in patients with advanced HCC but its failure in some patients poses a challenge for clinicians. Four trials demonstrated the efficacy of three drugs for the population with HCC unresponsive to sorafenib: RESORCE (regorafenib), CELESTIAL (cabozantinib), REACH, and REACH-2 (ramucirumab) trials. The therapeutic window has been further widened by immunotherapy, including nivolumab alone or in combination with ipilimumab or pembrolizumab, which has received FDA approval [13][14][15]. However, the choice of a second-line treatment is still left to clinical judgment rather than being evidence-based, due to a lack of head-to-head trials. To address this deficit, a meta-analysis has been conducted to detect potential differences among distinctive subgroups of patients that could influence therapeutic decision-making. Moreover, it has been examined whether disease progression despite sorafenib treatment and/or sorafenib intolerance impacted the selection of a second drug.

To our knowledge, only Bakouny et al. explored the second-line treatment options in HCC [32]. These analyses substantially extend those findings, by adding data available from clinical trials, including those reporting the therapeutic efficacy but also the safety of immunotherapy. Additionally, the patient cohort was stratified solely according to the second-line therapy, when possible.

This network meta-analysis showed that regorafenib had the strongest effect in prolonging OS and also had an acceptable safety profile. However, the efficacy of regorafenib therapy was demonstrated only in patients with disease progression despite sorafenib therapy, while patients with sorafenib intolerance (which accounts for up to half of the cases of sorafenib failure seen in daily clinical practice [33] were excluded from the trial. Conversely, sorafenib intolerance was not an exclusion criterion for recruitment into the CELESTIAL trial, such that cabozantinib remains an alternative for sorafenib-intolerant patients. Unfortunately, as CELESTIAL did not report outcomes stratified for the sorafenib response, statistical proof of the efficacy of cabozantinib in this subset of patients is lacking, and in case of benefit the extent could not be estimated. The pooled analysis of REACH and REACH-2 showed that ramucirumab is effective in patients with disease progression despite sorafenib therapy but ineffective in sorafenib-intolerant patients. An OS stratified for the response to sorafenib was available for ADIPEG20, brivanib, everolimus, pembrolizumab, ramucirumab, and tivantinib. Of these, the statistically significant efficacy of regorafenib, ramucirumab, and pembrolizumab in prolonging OS compared to placebo was demonstrated, but not in sorafenib-intolerant patients. However, the sample size of the sorafenib-intolerant subgroup might has been statistically underpowered. The lack of data on alternative therapies for sorafenib-intolerant patients is problematic and for the clinician with a patient in whom sorafenib has failed, the best second-line treatment remains a black box.

In fact, data are lacking for nearly every subgroup of patients, despite the current emphasis on patient-tailored therapy [34]. In the treatment of any disease, age, biological, and pathological characteristics must be considered along with treatment-related features. Information on the overall efficacy of a drug may be of little value when its efficacy in a specific subgroup of patients is unknown. Moreover, this meta-analysis showed a lack of robust evidence able to guide the choice of one treatment over an alternative one, irrespective of the specific subgroup. Statistically powered clinical trials aimed at evaluating patient-tailored second-line options and devised based on specific hypotheses are needed.

An example is provided by the REACH trial, which demonstrated the efficacy of ramucirumab for patients with high AFP levels, and then confirmed and expanded this result in REACH-2. In REACH, ramucirumab was not effective in the low AFP subgroup, despite the overall benefit determined in the REACH and REACH-2 pooled analysis. Thus, the inclusion of patients with low AFP levels masked the benefits of the drug afforded to the subgroup of patients with high AFP levels. In fact, according to the NCCN guideline on HCC, ramucirumab is a choice only in patients with AFP levels ≥ 400 ng/mL. A related issue is the under-representation of females in clinical trials, although this parallels the male predominance in HCC. Overall, males seem to better respond to second-line treatments but the underlying reasons and variables remain to be elucidated. Nonetheless, both literature data and this analysis point toward a slightly decreased therapeutic efficacy of immune checkpoint inhibitors [35] as well as a trend toward a higher effectiveness of tivantinib in females. The finding of a higher efficacy of pembrolizumab in patients with HBV infection, low AFP levels, macrovascular invasion, and no extrahepatic spread also suggests differences depending on the biological landscape. It would be worth to deeper explore the potential biology beyond the gender characteristic.

Owing to the lack of direct evidence from RCTs, in the present study an adjusted indirect comparison was adopted as a surrogate. To minimize the potential risk of bias, a mirror principle was applied to ensure the internal similarity of the included studies. However, head-to-head studies are warranted for direct comparisons of alternative interventions.

## Conclusions

The findings of this systematic review and meta-analysis indicate that regorafenib and cabozantinib represent two established options for second-line treatment of HCC in overall population. They also highlight key methodological gaps in the available trials that challenge the unequivocal identification of the best therapeutic option when patients are stratified by subgroups. Future studies are required to address this issue by taking into account the tumor biology and the patient clinical characteristics.

## Supplementary Information

Below is the link to the electronic supplementary material.

Supplementary file1 (PDF 2367 kb)

## Abbreviations

HCC: Hepatocellular carcinoma
OS: Overall survival
PFS: Progression-free survival
RCTs: Randomized controlled trials
HR: Hazard ratio
CI: Confidence interval
BCLC: Barcelona Clinic Liver Cancer
ECOG PS: Eastern Cooperative Oncology Group Performance Status
FDA: Food and Drug Administration
BSC: Best supportive care
PD-1: Programmed death-1
PRISMA: Preferred Reporting Items for Systematic Reviews and Meta-Analyses
ORs: Odds ratio
AFP: Alpha-fetoprotein
