# Supplementary material for: Second-line treatments for Advanced Hepatocellular Carcinoma: A Systematic Review and Bayesian Network Meta-analysis
Source: Clin Exp Med. 2021 Jun 19;22(1):65–74. doi: 10.1007/s10238-021-00727-7 (PMC8863772; doi:10.1007/s10238-021-00727-7)

Supplementary Figure 1: Network graph.

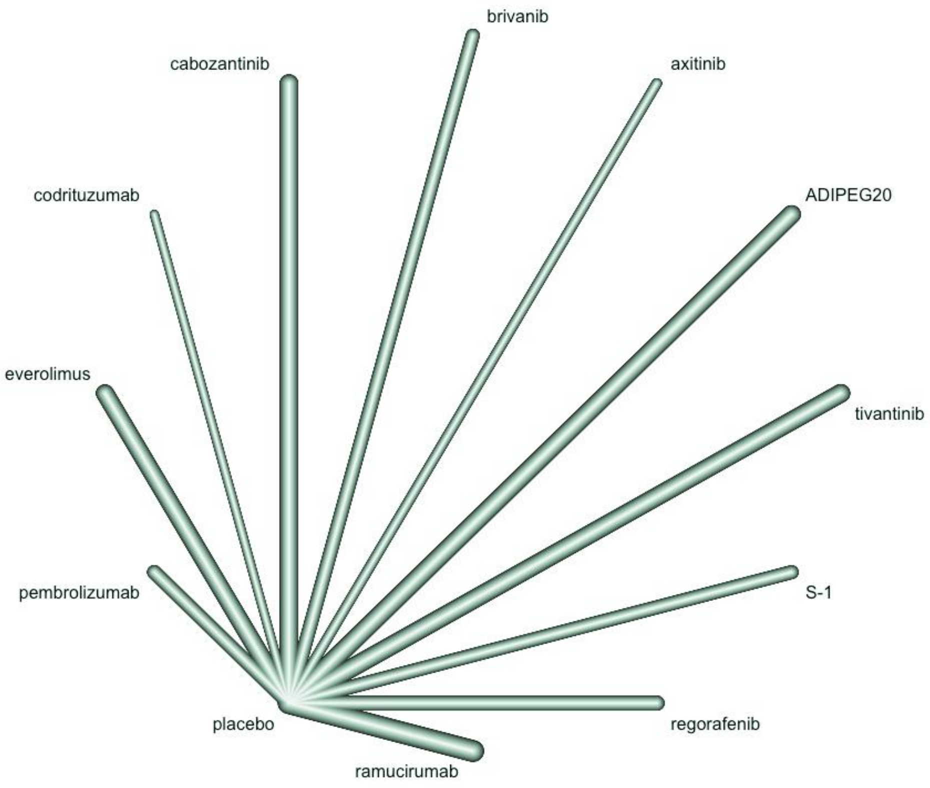

Supplementary Figure 2: Forest plot of overall survival. The comparisons were made against regorafenib.

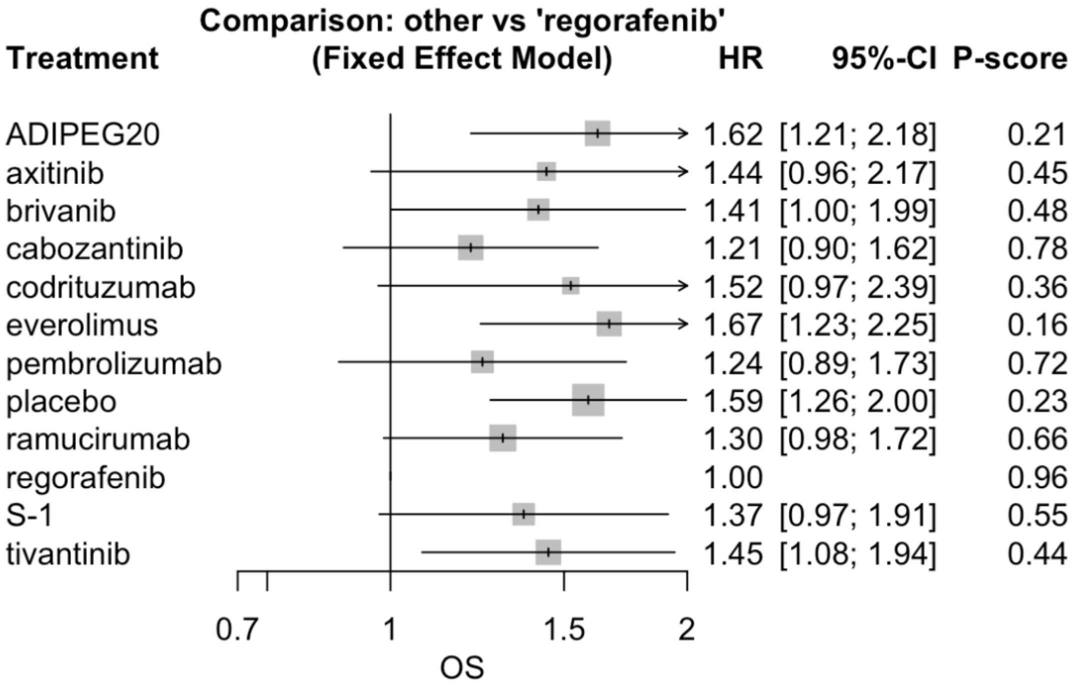

Supplementary Figure 3: Forest plot of progression free survival. The comparisons were made against regorafenib.

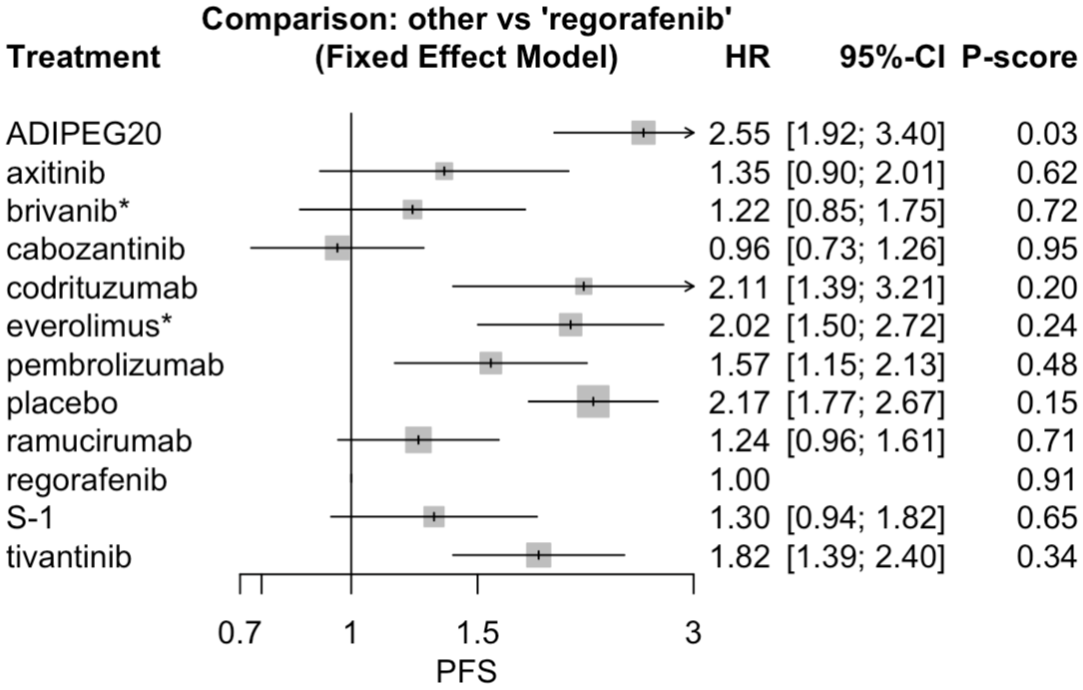

Supplementary Figure 4: A: Forest plot of overall survival in the subgroup of sorafenib-intolerant patients. The comparisons were made against the placebo; B: Forest plot of overall survival in the subgroup of sorafenib-refractory patients. The comparisons were made against the placebo.

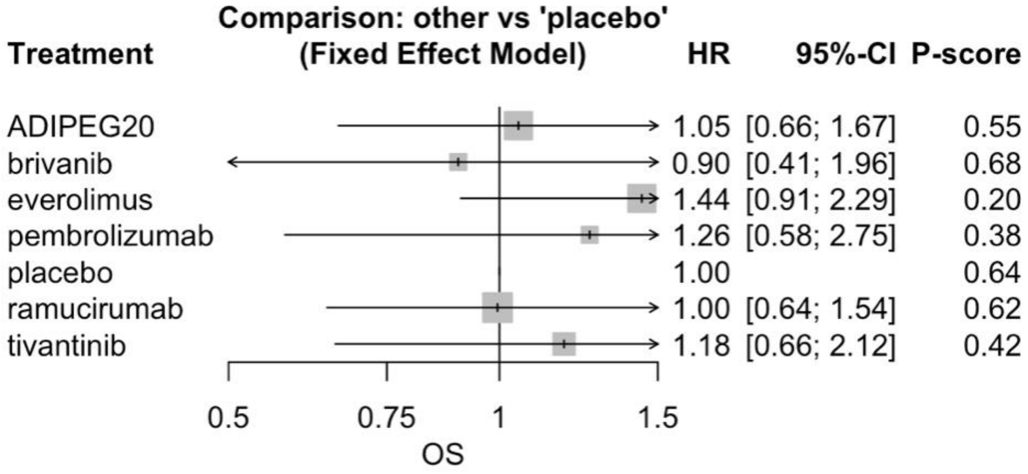

A

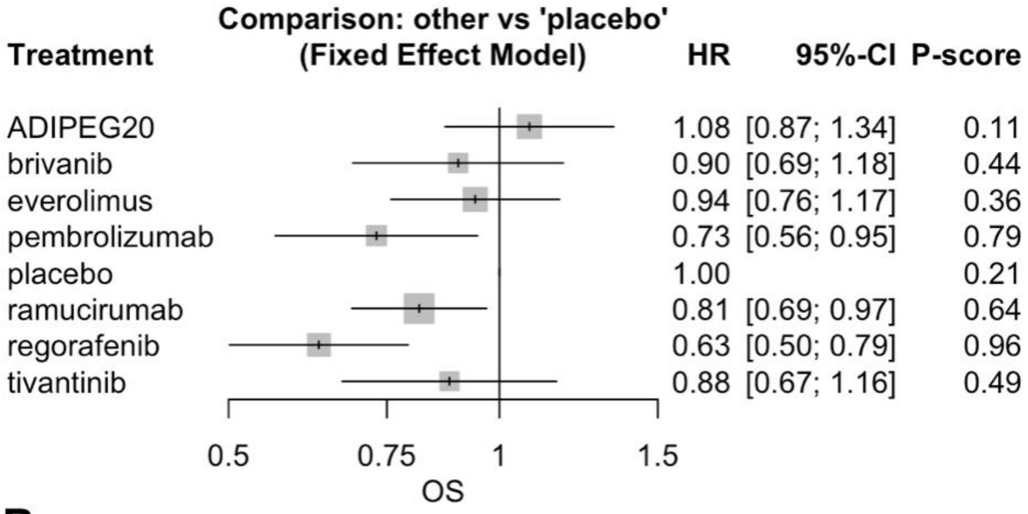

B

Supplementary Table 1: Population characteristics across the clinical trials.

Study name	Male gender	Female gender	Age	ECOG 0	Extra-hepatic spread	Macrovascular invasion	High AFP	Sorafenib intolerance	Sorafenib progression
BRISK-PS	82%/86%	18%/14%	64/62	57%/61%	65%/64% (M)	31%/18%	50%/44%	13%/12%	86%/88%
Santoro A. et al.	82%/78%	18%/22%	69-71/68[1]	58%/58%	61%/78%	69%/64%	44%/58%	-	-
EVOLVE-1	83,7%/84%	16,3%/16%	67/64	59,1%/56,5%	74,3%/73,4%	32,9%/32,6%	47,2%/47,8%	18,5%/20,1%	81,2%/79,9%
Kang Y.-K. et al.	82%/82%	18%/18%	61/63	58%/57%	69%/71%	25%/28%	-	-	-
REACH	83%/86%	17%/14%	64/62	56%/54%	73%/71%	29%/28%	42%/46%	13%/15%	87%/85%
Abou-Aifa G.K. et al.	85%/75%	15%/25%	64/62,5	65%/63%	-	-	-	-	-
RESORCE	88%/88%	12%/12%	64/62	65%/67%	70%/76%	29%/28%	43%/45%	0%/0%	100%/100%
S-CUBE	82%/77%	18%/23%	70/70	85%/81%	55%/52% (M)	16%/21%	41%/44%	34%/31%	66%/69%
Abou-Aifa G. et al.	83%/80%	17%/20%	61/62	-	-	-	49,5%/50,7%	16%/17%	70%/69%
CELESTIAL	81%/85%	19%/15%	64/64	52%/55%	79%/77%	27%/34%	41%/43%	-	-
Metiv-HCC	88%/94%	12%/6%	66/65	62%/58%	58%/59%	35%/33%	43%/42%	17%/21%	82%/78%
REACH 2	78%/83%	22%/17%	64/64	57%/58%	72%/74%	36%/35%	100%/100%	16%/20%	84%/80%
JET-HCC	82,1%/85,2%	17,9%/14,8%	70/72	82,8%/83,6%	-	25,4%/27,9%	-	14,9%/23%	85,1%/77%
KEYNOTE-240	81,3%/83%	18,7%/17%	67/65	58,3%/52,6%	70,1%/68,9%	12,9%/11,9%	46,4%/43%	12,9%/13,3%	87,1%/86,7%

1 Three active arms

Study name	HBV	HCV	Alcohol	Asian	Black	White	Comments
BRISK-PS	39%/34%	28%/27%	23%/27%	48%/45%	4%/5%	46%/50%	
Santoro A. et al.	23%/14%	51%/39%	-	4%/6%	6%/3%	89%/92%	
EVOLVE-1	25,1%/28,3%	26%/23,4%	17,7%/24,5%	37,8%/31,5%	1,7%/1,6%	53%/59,8%	
Kang Y.-K. et al.	51%/50%	29%/16%	-	63%/62%	1%/0%	36%/38%	Only 95%/85% of patients exposed to sorafenib containing regimen as first line
REACH	35%/36%	27%/27%	-	46%/48%	-	49%/49%	
Abou-Aifa G.K. et al.	38%/42%	27%/30%	-	46%/42%	-	46%/55%	Only 84%/70% of patients exposed to sorafenib containing regimen as first line
RESORCE	38%/38%	21%/21%	24%/28%	41%/40%	2%/1%	36%/35%	Only patients with sorafenib-progression included
S-CUBE	20%/23%	55%/53%	-	-	-	-	
Abou-Aifa G. et al.	53%/50%	26%/26%	12%/15%	53%/53%	-	-	
CELESTIAL	38%/38%	24%/23%	24%/16%	34%/35%	2%/5%	56%/55%	
Metiv-HCC	18%/18%	32%/29%	-	4%/6%	5%/1%	72%/75%	
REACH 2	36%/38%	24%/29%	24%/22%	52%/47%	-	30%/33%	Only patients with elevated AFP included
JET-HCC	26,1%/26,2%	45,5%/50,8%	20,1%/23%	100%/100%	0%/0%	0%/0%	Only japanese patients included
KEYNOTE-240	25,9%/21,5%	15,5%/15,6%	57,2%/58,5%	-	-	-	

Supplementary Table 2: Risk of bias assessment.

Study name	Study name	Selection bias	Reporting bias	Performance bias	Detection bias	Attrition bias
BRISK-PS (18)	BRISK-PS	Unclear	High	Low	Low	Low
Santoro A. et al. (24)	Tivantinib	Low	Low	Low	Low	Low
EVOLVE-1 (21)	EVOLVE-1	Unclear	Low	High	High	Low
Kang Y.-K. et al. (25)	Axitinib	Unclear	High	Low	High	Low
REACH (26)	REACH	Unclear	Low	High	High	Low
Abou-Aifa G.K. et al. (29)	Codrituzumab	Unclear	High	High	High	Low
RESORCE (16)	RESORCE	Unclear	Low	Low	Low	Low
S-CUBE (30)	S-CUBE	Unclear	High	Low	High	Low
Abou-Aifa G. et al. (20)	ADIPEG20	Unclear	Low	Low	Low	Low
CELESTIAL (19)	CELESTIAL	Unclear	High	Low	Low	Low
Metiv-HCC (22)	METIV-HCC	Low	Low	Low	Low	Low
REACH 2 (27)	REACH 2	Unclear	Low	High	High	Low
JET-HCC (28)	JET-HCC	High	High	Low	Low	Low
KEYNOTE-240 (23)	KEYNOTE-240	Unclear	Low	High	Low	Low

Supplementary Table 3: Secondary outcomes.

**Study name**	BRISK-PS (18)	Santoro A. et al. (24)	EVOLVE-1 (21)	Kang Y.-K. et al. (25)	REACH (26)	Abou-Aifa G.K. et al. (29)	RESORCE (16)
Age							
Low	-	-	0.95 (0.72-1.26)	0.72 (0.46-1.13)	0.92 (0.71-1.18)	-	0.65 (0.49-0.87)
High	-	-	1.10 (0.84-1.45)	1.09 (0.66-1.82)	0.83 (0.62-1.11)	-	0.74 (0.54-1.02)
Gender							
Male	-	-	-	0.91 (0.63-1.13)	0.88 (0.71-1.08)	-	0.65 (0.52-0.82)
Female	-	-	-	0.83 (0.36-1.93)	0.70 (0.42-1.16)	-	0.88 (0.48-1.62)
ECOG							
0	0.90 (0.70-1.30)	0.82 (0.44-1.50)	1.02 (0.79-1.33)	1.08 (0.68-1.73)	0.80 (0.61-1.04)	-	0.61 (0.47-0.80)
>0	0.80 (0.50-1.10)	0.98 (0.50-1.93)	1.03 (0.77-1.39)	0.60 (0.37-0.97)	1.02 (0.77-1.35)	-	0.78 (0.55-1.11)
Extra-hepatic spread							
Yes	-	1.04 (0.61-1.76)	1.02 (0.81-1.27)	-	0.79 (0.63-0.98)	-	0.60 (0.47-0.77)
No	-	0.45 (0.18-1.12)	1.05 (0.71-1.54)	-	1.22 (0.83-1.79)	-	0.97 (0.63-1.48)
Macrovascular invasion							
Yes	-	1.89 (0.85-4.20)	1.21 (0.87-1.68)	0.98 (0.52-1.86)	0.72 (0.50-1.03)	-	0.67 (0.46-0.98)
No	-	0.60 (0.35-1.04)	0.87 (0.54-1.14)	0.87 (0.59-1.29)	0.86 (0.68-1.08)	-	0.67 (0.52-0.86)
AFP							
Low	0.70 (0.50-1.00)	1.01 (0.51-1.97)	0.98 (0.73-1.32)	1.27 (0.76-2.12)	1.09 (0.84-1.43)	-	0.67 (0.50-0.90)
High	1.00 (0.70-1.30)	0.77 (0.42-1.44)	1.20 (0.91-1.57)	0.67 (0.42-1.07)	0.67 (0.51-0.90)	-	0.68 (0.50-0.92)
Sorafenib response							
Intolerance	0.90 (0.40-1.90)	-	1.44 (0.91-2.30)	-	1.36 (0.77-2.39)	-	-
Progression	0.90 (0.70-1.20)	-	0.94 (0.76-1.17)	-	0.84 (0.68-1.03)	-	0.63 (0.50-0.79)
Infection status							
HBV	1.00 (0.70-1.50)	0.99 (0.32-3.04)	0.64 (0.45-0.93)	-	0.79 (0.58-1.07)	-	0.58 (0.41-0.82)
HCV	0.80 (0.50-1.30)	0.65 (0.32-1.32)	0.96 (0.65-1.43)	-	0.88 (0.61-1.26)	-	0.79 (0.49-1.26)
No infection	-	0.87 (0.42-1.79)	1.35 (1.01-1.80)	-	0.95 (0.69-1.30)	-	-

Supplementary Table 3: Secondary outcomes – Continued.

**Study name**	S-CUBE (30)	Abou-Aifa G. et al. (20)	CELESTIAL (19)	Metiv-HCC (22)	REACH 2 (27)	JET-HCC (28)	KEYNOTE-240 (23)
Age							
Low	-	-	0.81 (0.62–1.05)	0.88 (0.62–1.27)	0.85 (0.57–1.27)	-	0.75 (0.52–1.09)
High	-	-	0.74 (0.56–0.97)	1.00 (0.71–1.43)	0.64 (0.43–0.96)	-	0.92 (0.65–1.30)
Sex							
Male	-	1.01 (0.83–1.23)	0.79 (0.64–0.97)	0.99 (0.76–1.28)	0.70 (0.51–0.95)	-	0.76 (0.58–1.00)
Female	-	1.04 (0.68–1.70)	0.68 (0.42–1.09)	0.57 (0.23–1.45)	1.21 (0.59–2.46)	-	0.80 (0.44–1.47)
ECOG							
0	-	-	0.69 (0.53–0.89)	0.99 (0.71–1.38)	0.71 (0.49–1.04)	0.90 (0.62–1.30)	0.73 (0.52–1.03)
>0	-	-	0.87 (0.66–1.14)	0.97 (0.65–1.43)	0.77 (0.50–1.18)	0.47 (0.21–1.08)	0.95 (0.66–1.37)
Extra-hepatic spread							
Yes	-	1.05 (0.85–1.29)	0.72 (0.58–0.89)	1.09 (0.78–1.52)	0.70 (0.51–0.98)	0.90 (0.58–1.40)	0.86 (0.64–1.16)
No	-	0.93 (0.64–1.34)	0.96 (0.63–1.46)	0.80 (0.54–1.18)	0.84 (0.48–1.48)	0.70 (0.40–1.23)	0.67 (0.42–1.08)
Macrovascular invasion							
Yes	-	1.28 (0.93–1.77)	0.75 (0.54–1.03)	1.19 (0.79–1.79)	0.97 (0.61–1.53)	0.77 (0.42–1.41)	0.57 (0.29–1.13)
No	-	0.95 (0.76–1.18)	0.80 (0.64–1.01)	0.89 (0.65–1.22)	0.60 (0.42–0.87)	0.91 (0.61–1.36)	0.82 (0.63–1.06)
AFP							
Low	-	-	0.81 (0.62–1.04)	1.00 (0.71–1.41)	-	-	0.68 (0.49–0.96)
High	-	-	0.71 (0.54–0.94)	0.83 (0.58–1.20)	0.71 (0.53–0.95)	-	0.88 (0.62–1.26)
Sorafenib response							
Intolerance	-	1.05 (0.66–1.66)	-	1.18 (0.66–2.13)	0.63 (0.32–1.26)	-	1.26 (0.58–2.76)
Progression	-	1.08 (0.87–1.34)	-	0.88 (0.67–1.16)	0.76 (0.56–1.04)	-	0.73 (0.56–0.94)
Infection status							
HBV	-	0.98 (0.76–1.26)	0.69 (0.51–0.94)	0.78 (0.44–1.37)	0.84 (0.52–1.35)	0.53 (0.25–1.11)	0.57 (0.35–0.94)
HCV	-	1.25 (0.85–1.80)	1.11 (0.72–1.71)	0.84 (0.53–1.33)	0.76 (0.44–1.33)	0.88 (0.52–1.50)	0.96 (0.48–1.92)
No infection	-	-	0.72 (0.54–0.96)	1.08 (0.76–1.55)	0.63 (0.38–1.06)	0.59 (0.29–1.21)	0.88 (0.64–1.20)

Appendix 1: Supplementary forest plots

1: Forest plot of overall survival for the high AFP subgroup. The comparisons were made against the placebo (A) and regorafenib (B).

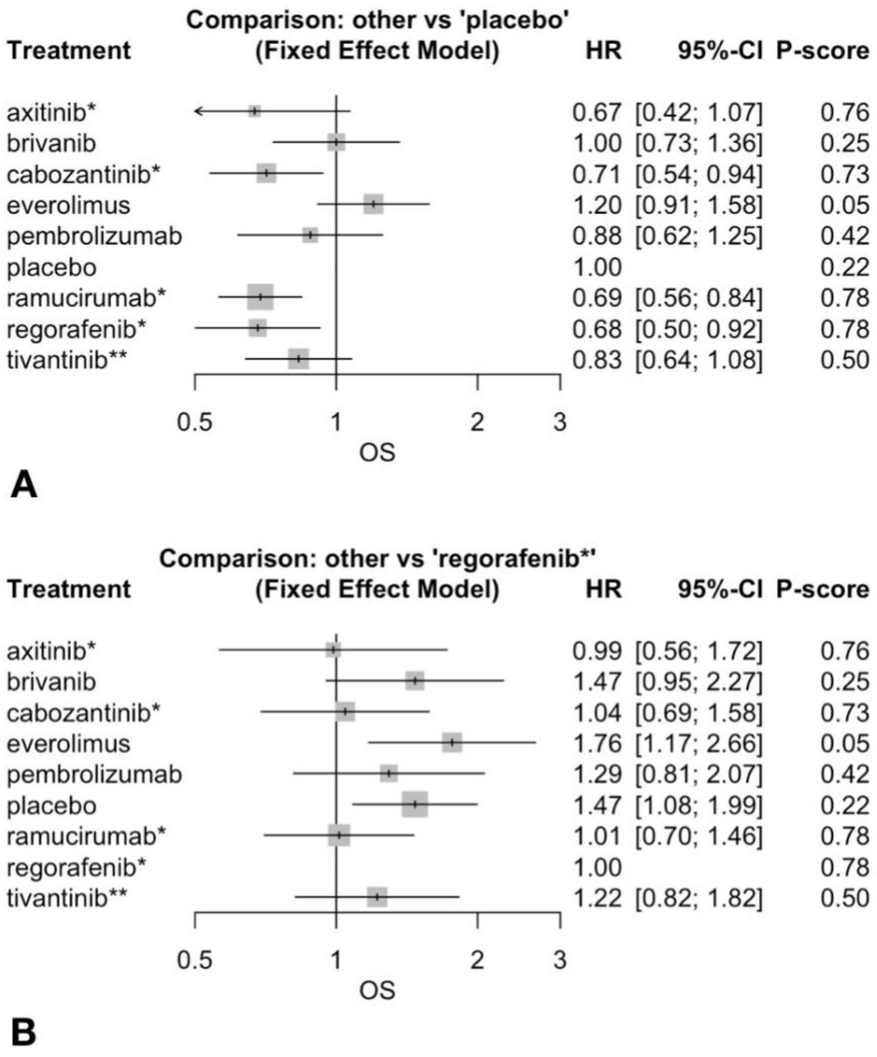

2: Forest plot of overall survival for the low AFP subgroup. The comparisons were made against the placebo (A) and regorafenib (B).

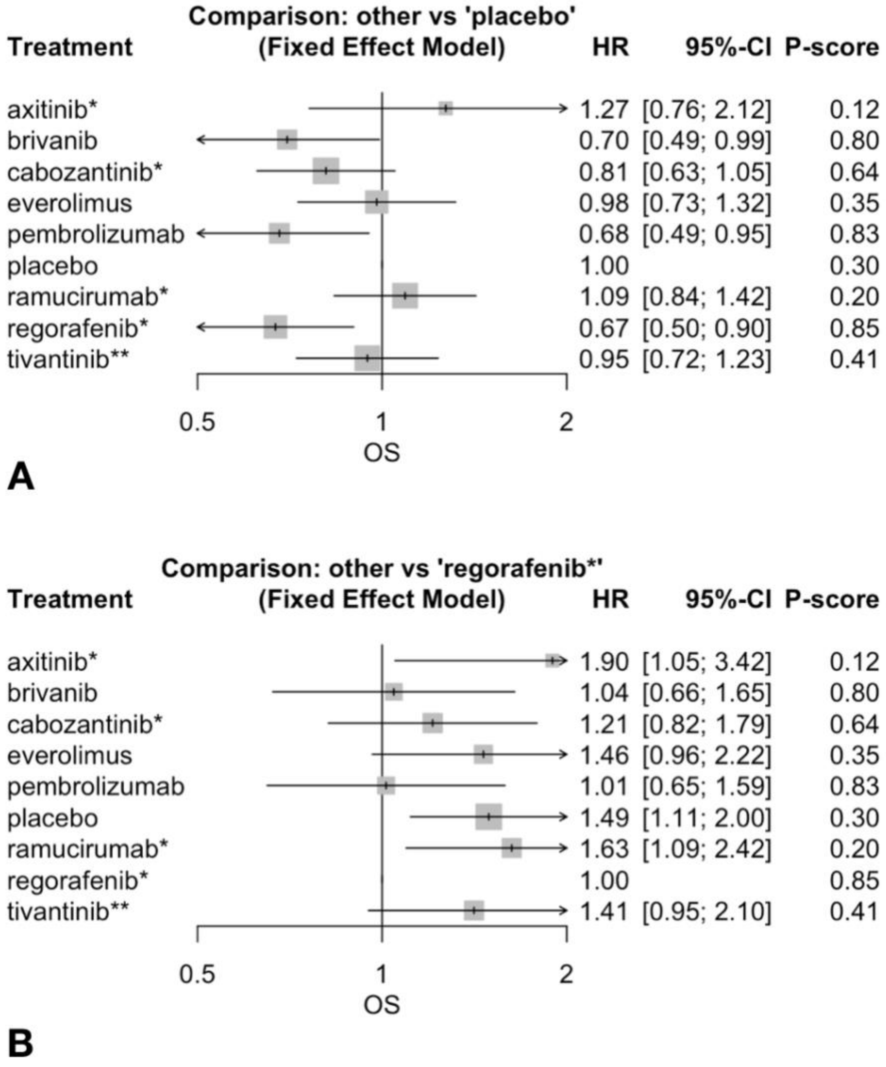

3: Forest plot of overall survival for the high age subgroup. The comparisons were made against the placebo (A) and regorafenib (B).

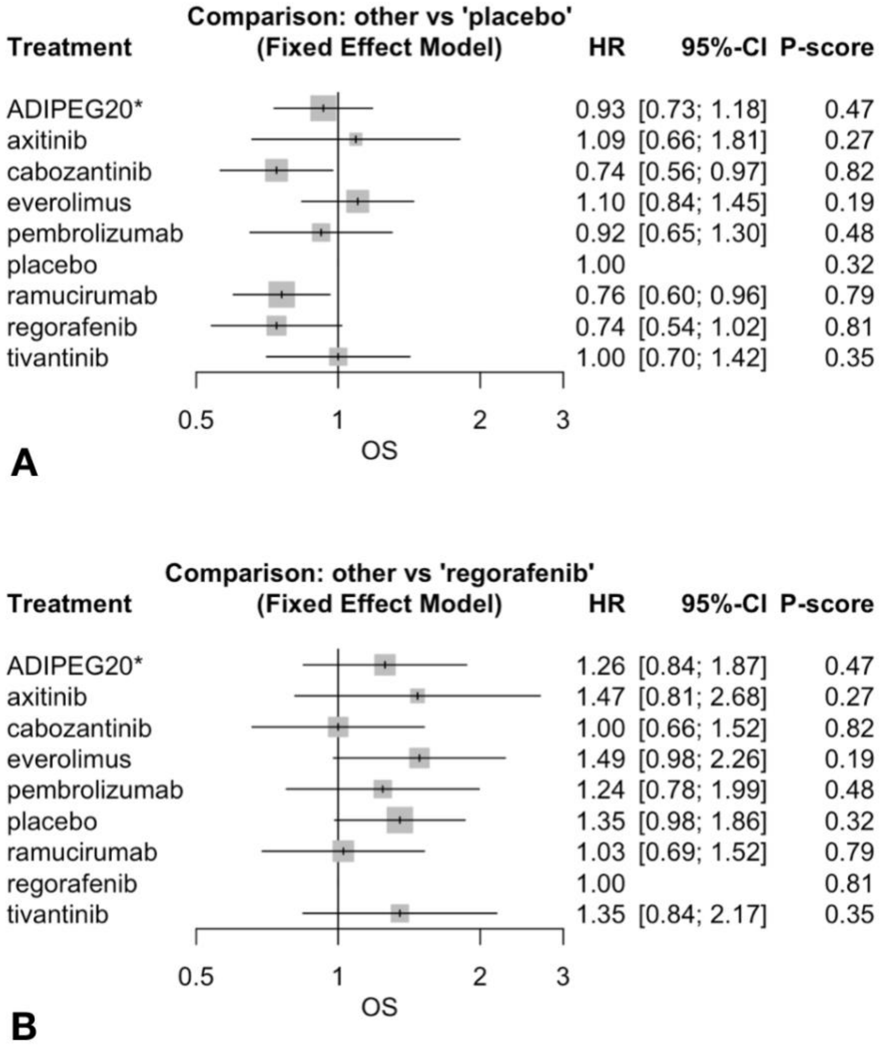

4: Forest plot of overall survival for the low age subgroup. The comparisons were made against the placebo (A) and regorafenib (B).

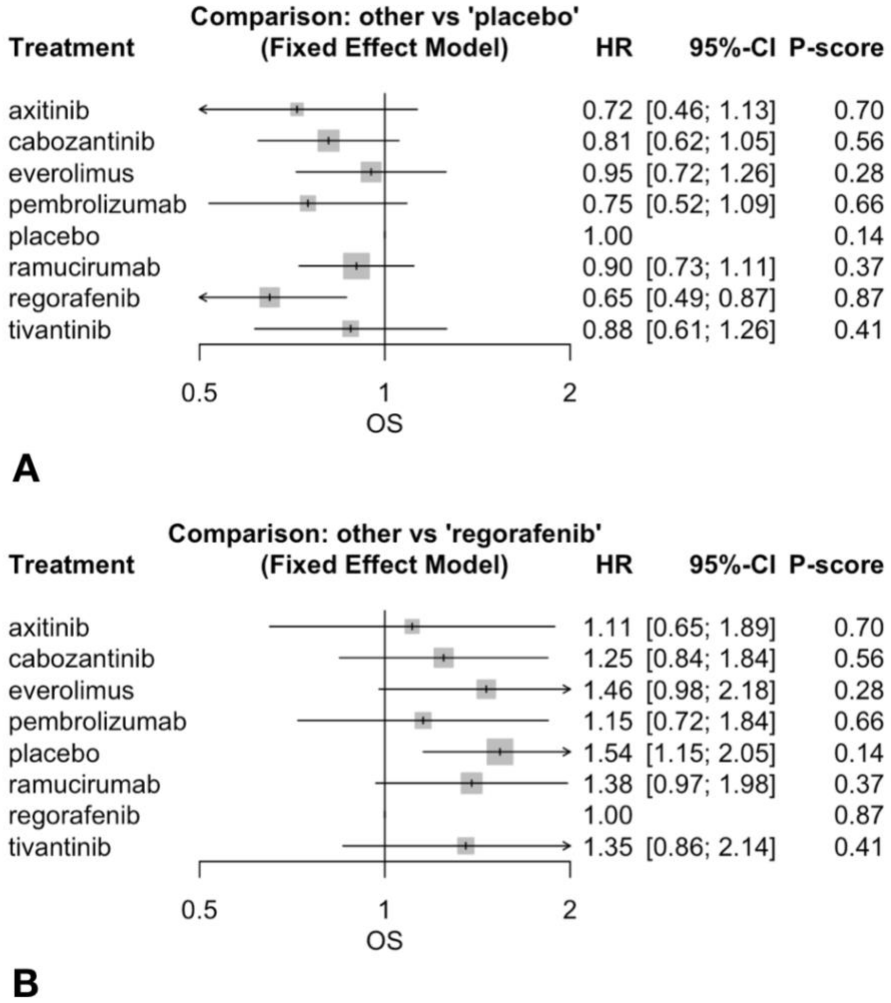

5: Forest plot of overall survival for the ECOG=0 subgroup. The comparisons were made against the placebo (A) and regorafenib (B).

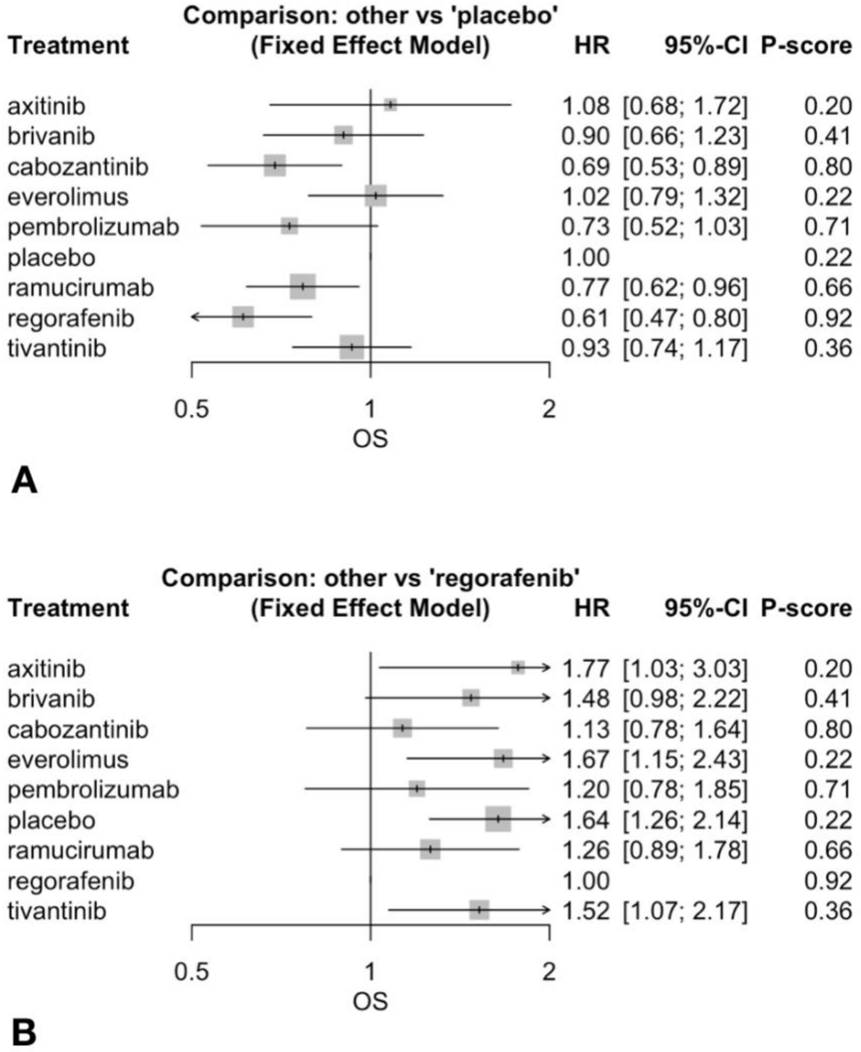

6: Forest plot of overall survival for the ECOG>0 subgroup. The comparisons were made against the placebo (A) and regorafenib (B).

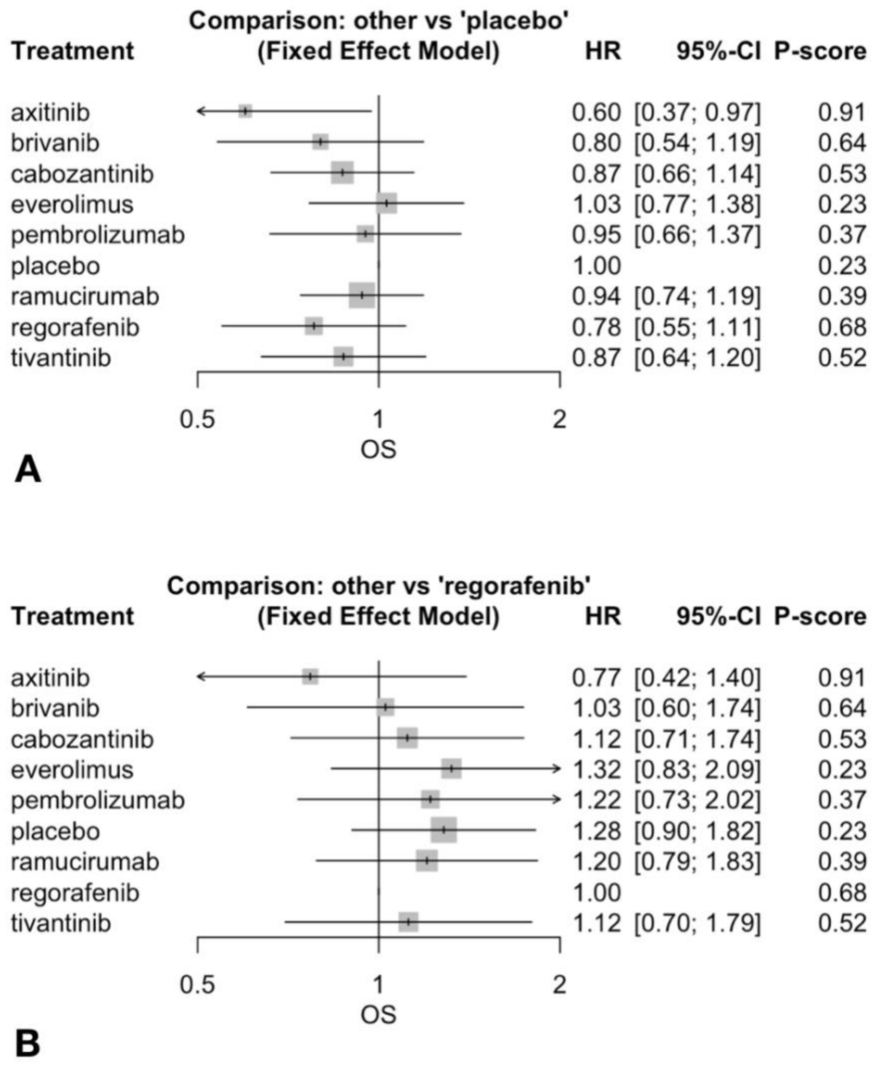

7: Forest plot of overall survival for the extrahepatic spread subgroup. The comparisons were made against the placebo (A) and regorafenib (B).

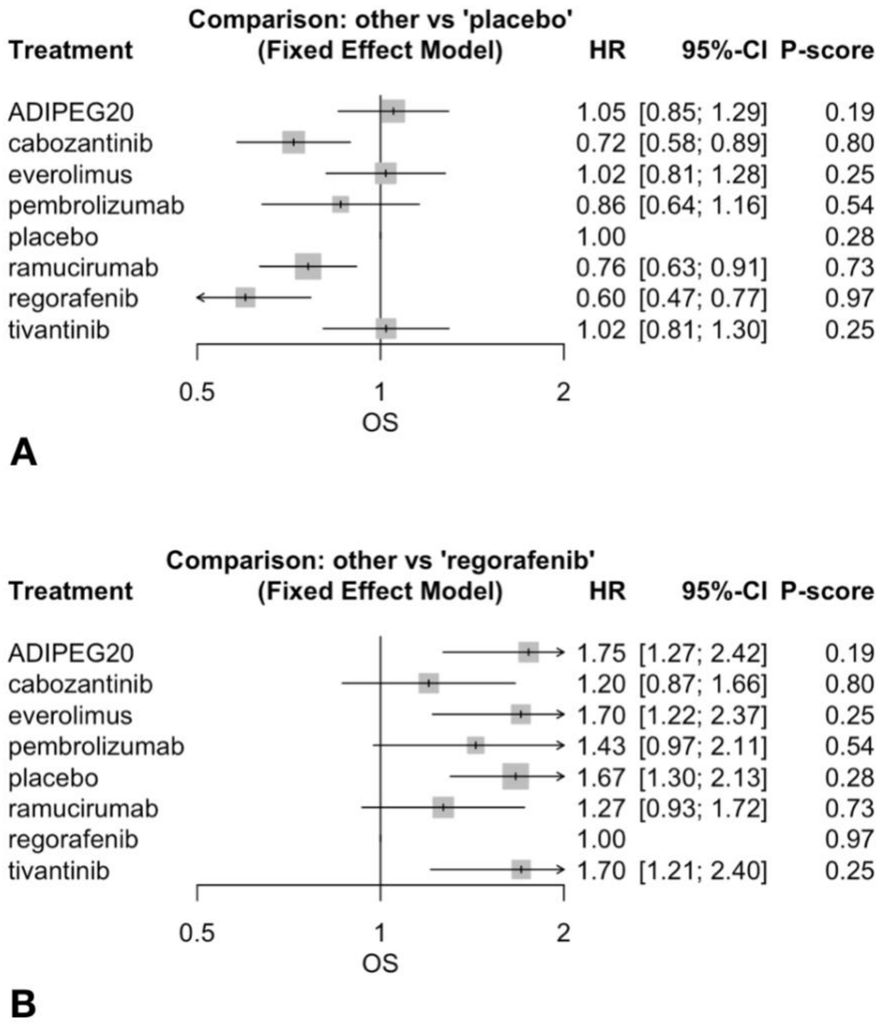

8: Forest plot of overall survival for the no-extrahepatic spread subgroup. The comparisons were made against the placebo (A) and regorafenib (B).

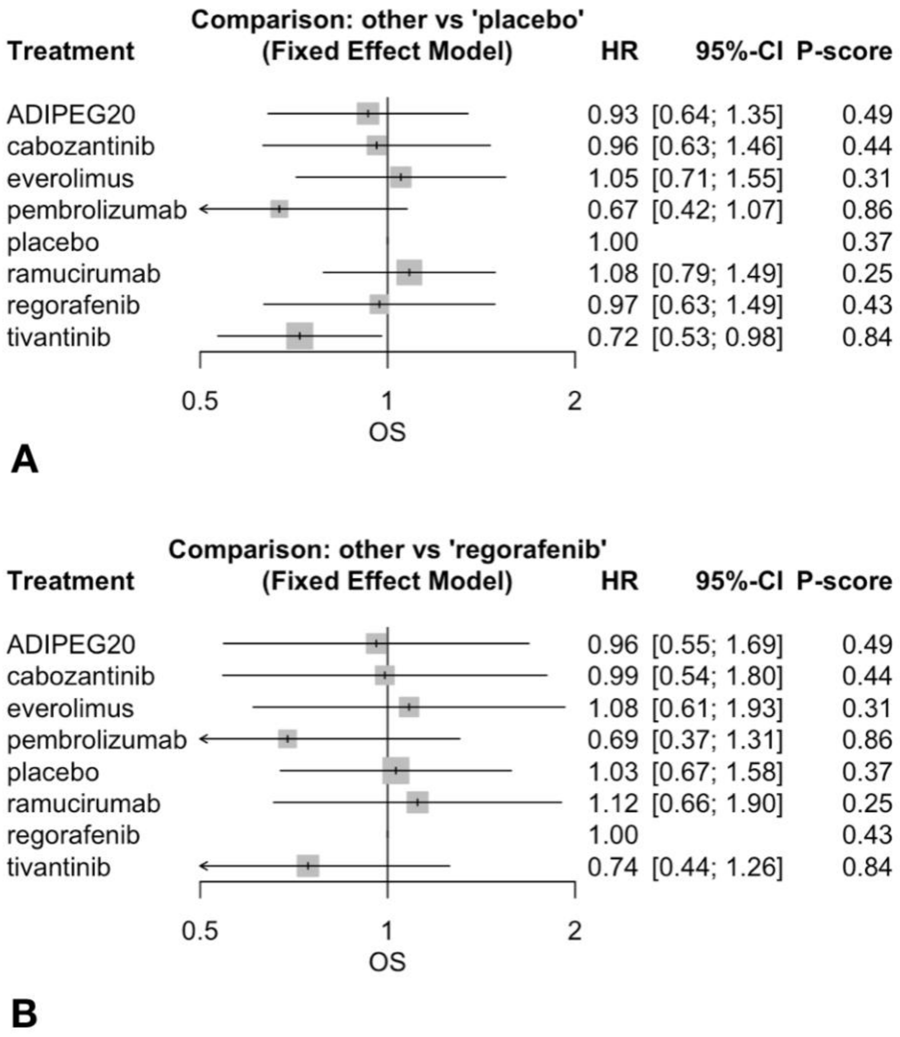

9: Forest plot of overall survival for the female gender subgroup. The comparisons were made against the placebo (A) and regorafenib (B).

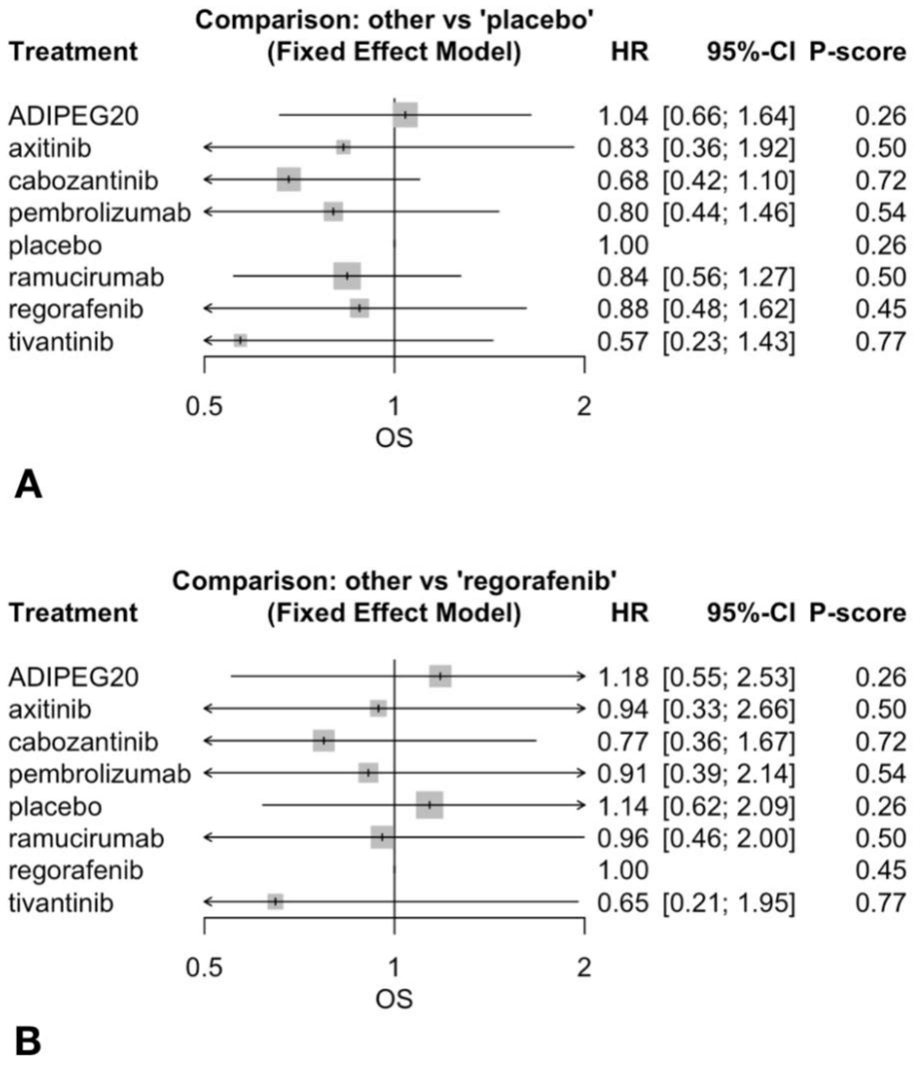

10: Forest plot of overall survival for the male gender subgroup. The comparisons were made against the placebo (A) and regorafenib (B).

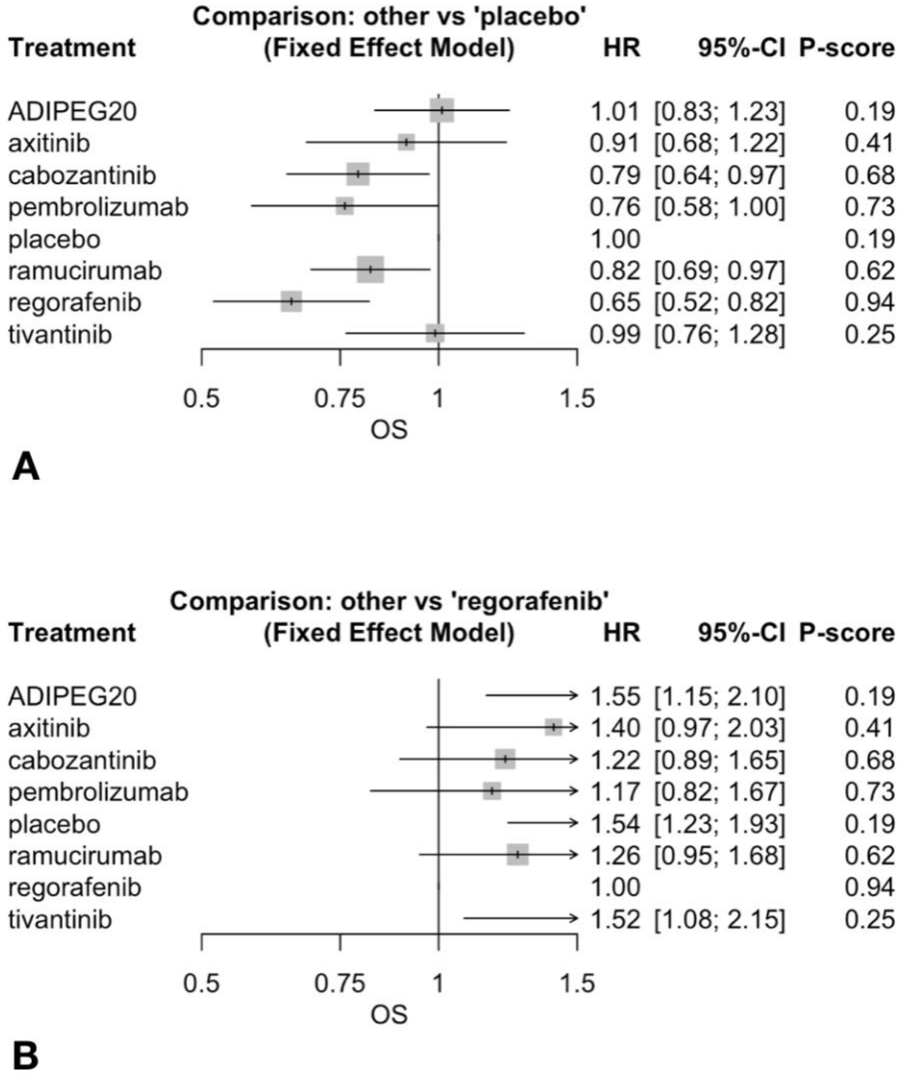

11: Forest plot of overall survival for the HBV subgroup. The comparisons were made against the placebo (A) and regorafenib (B).

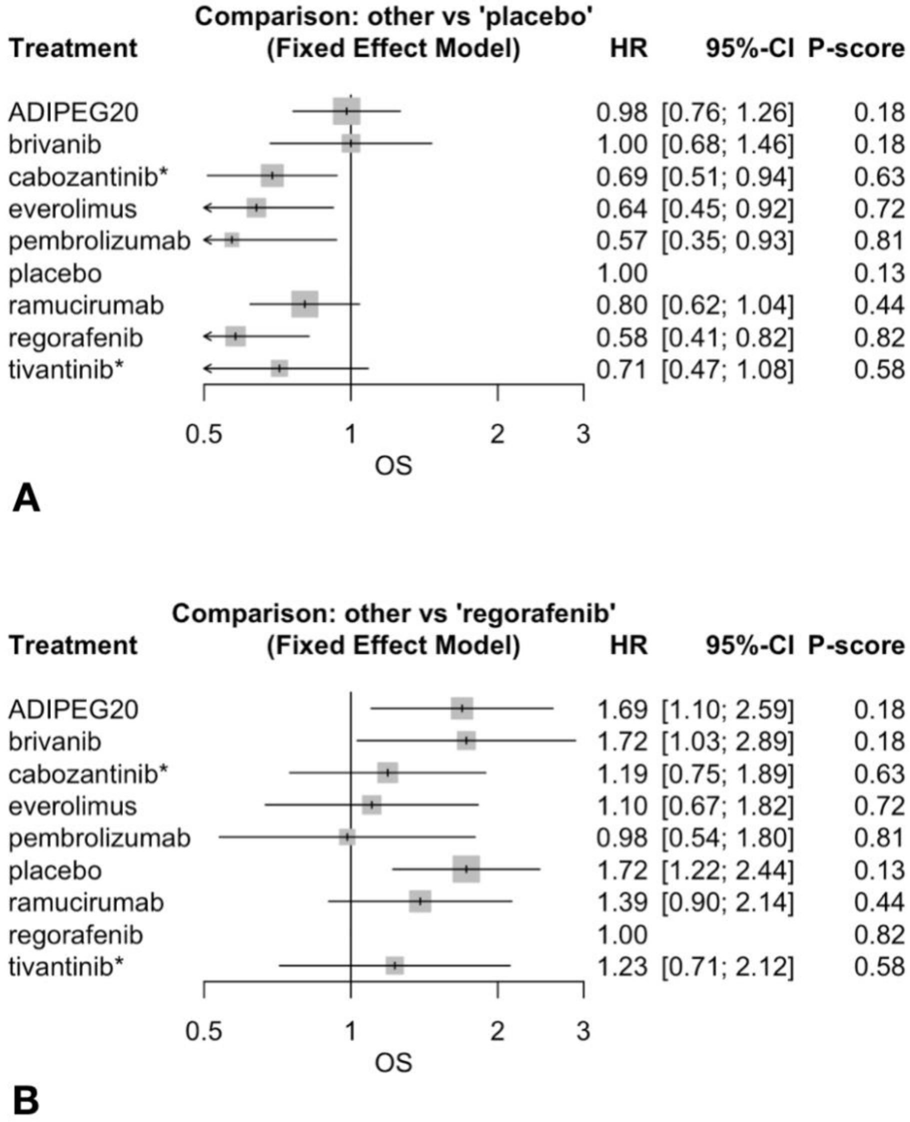

12: Forest plot of overall survival for the HCV subgroup. The comparisons were made against the placebo (A) and regorafenib (B).

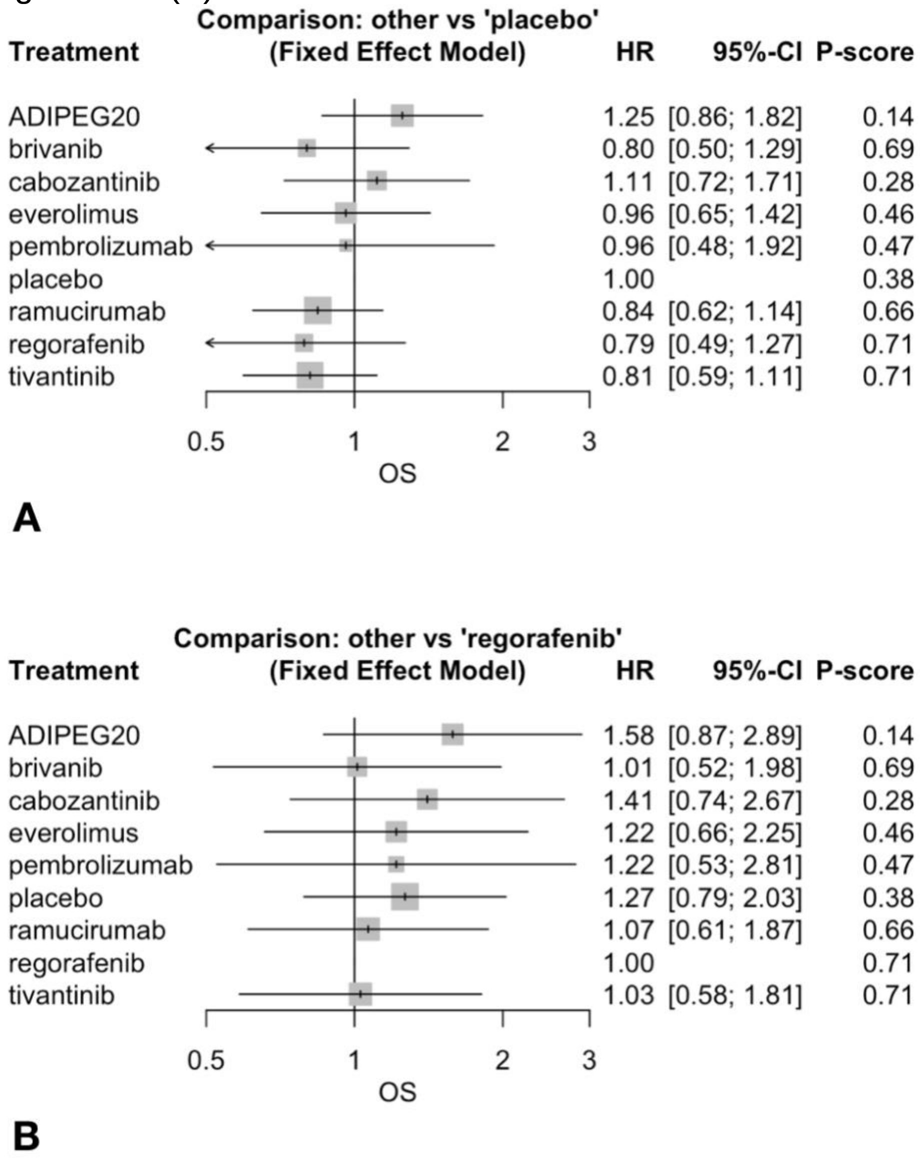

13: Forest plot of overall survival for the no-infection subgroup. The comparisons were made against the placebo.

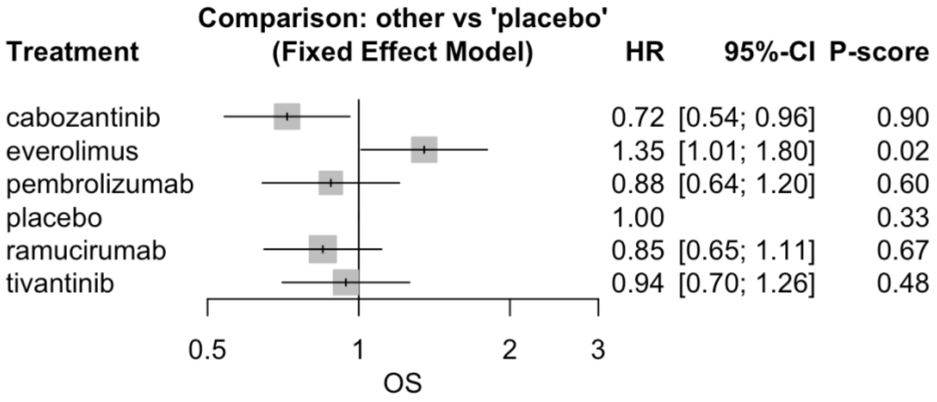

14: Forest plot of overall survival for the macrovascular invasion subgroup. The comparisons were made against the placebo (A) and regorafenib (B).

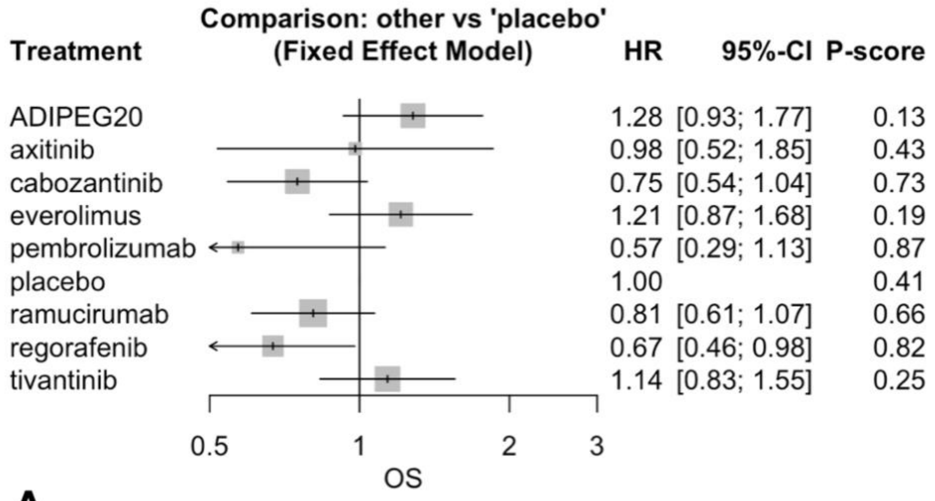

A

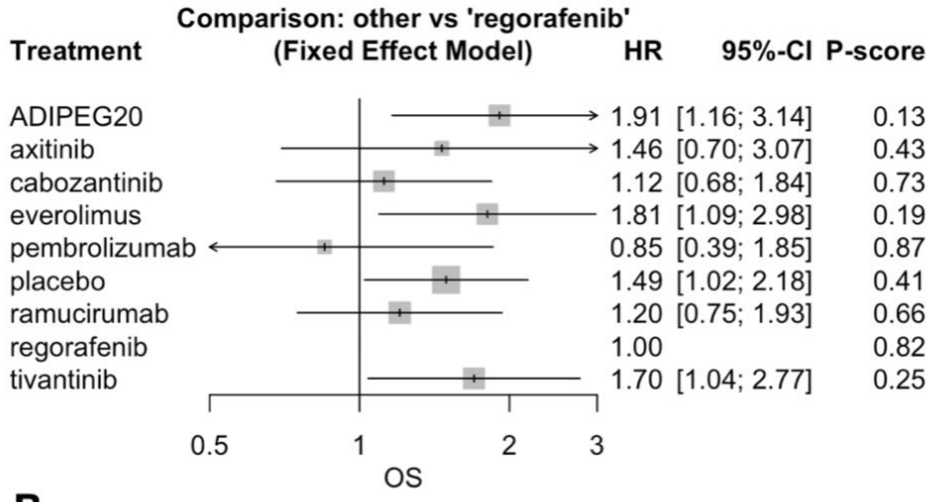

B

15: Forest plot of overall survival for the no-macrovascular invasion subgroup. The comparisons were made against the placebo (A) and regorafenib (B).

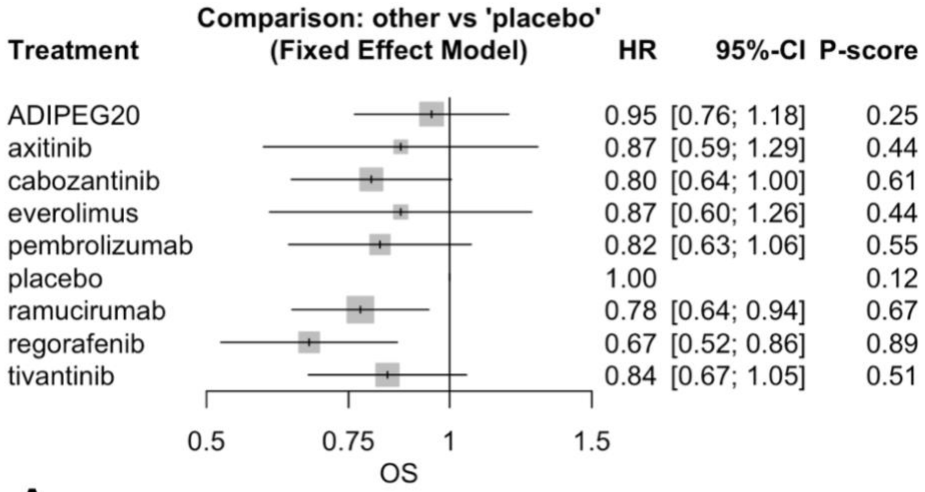

A

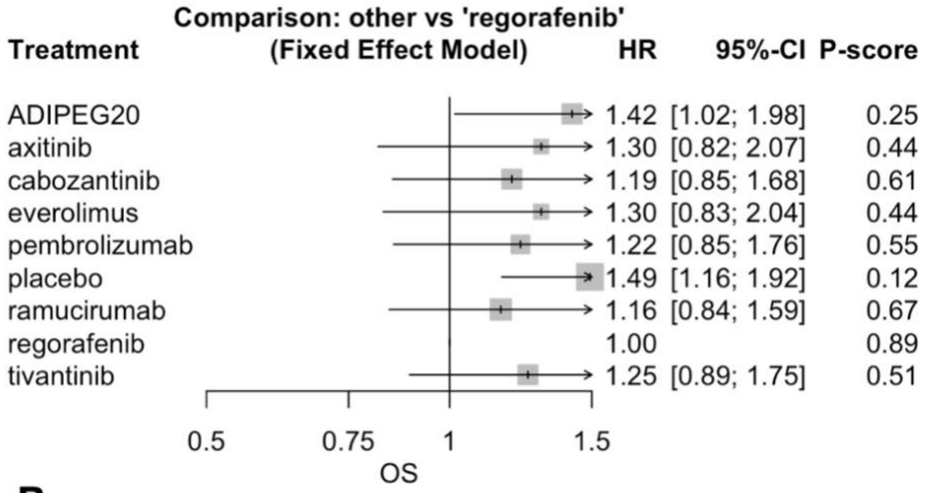

B

16: Forest plot of overall survival for the sorafenib-progression subgroup. The comparisons were made against the placebo (A) and regorafenib (B).

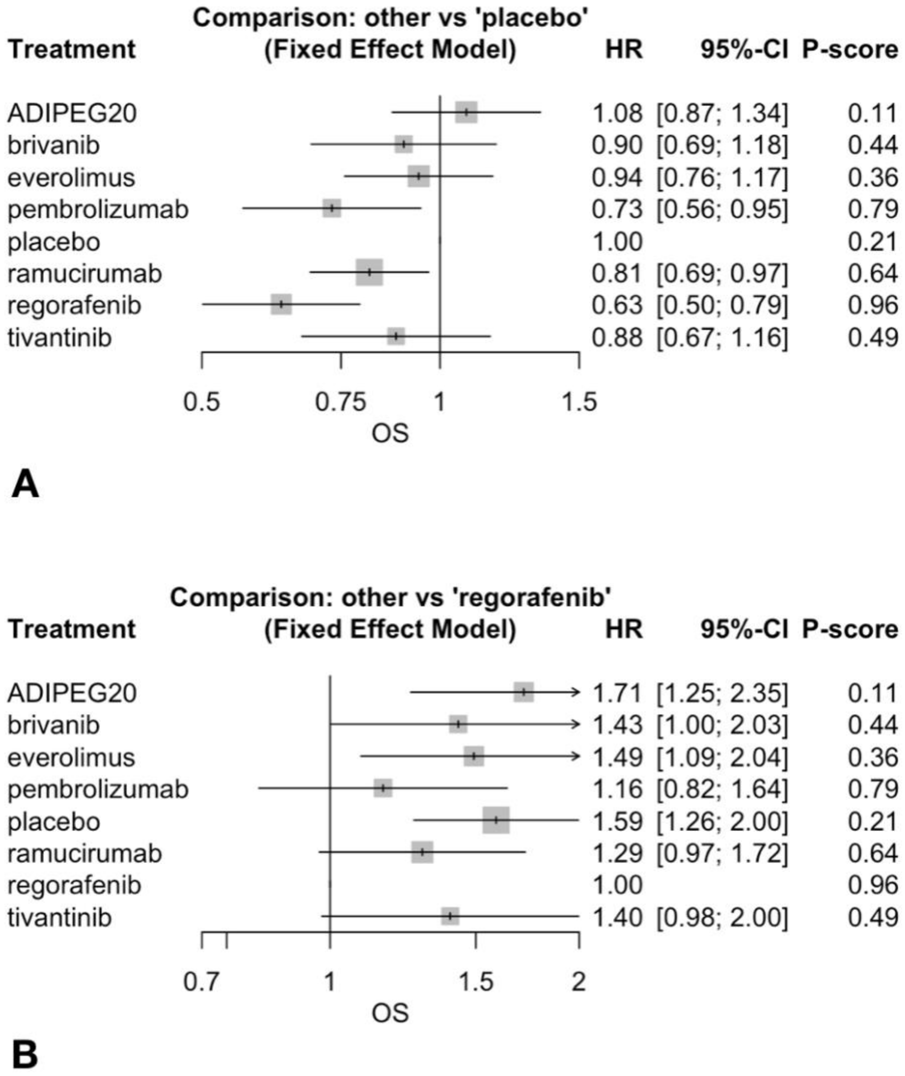

17: Forest plot of overall survival for the sorafenib-intolerance subgroup. The comparisons were made against the placebo.

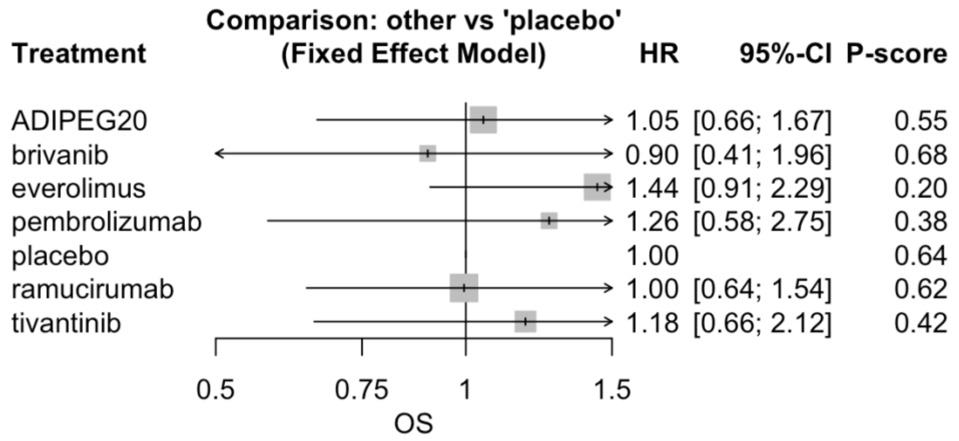

Supplement: Supplementary file 1 — Supplementary file1 (PDF 2367 kb) [file 10238_2021_727_MOESM1_ESM.pdf]
